# DNA replication initiation shapes the mutational landscape and expression of the human genome

**DOI:** 10.1126/sciadv.add3686

**Published:** 2022-11-09

**Authors:** Pierre Murat, Consuelo Perez, Alastair Crisp, Patrick van Eijk, Simon H. Reed, Guillaume Guilbaud, Julian E. Sale

**Affiliations:** ^1^ Division of Protein & Nucleic Acid Chemistry, MRC Laboratory of Molecular Biology, Francis Crick Avenue, Cambridge, CB2 0QH, UK.; ^2^ Broken String Biosciences Ltd., BioData Innovation Centre, Unit AB3-03, Level 3, Wellcome Genome Campus, Hinxton, Cambridge CB10 1DR, UK.; ^3^ Division of Cancer & Genetics School of Medicine, Cardiff University, Heath Park, Cardiff CF14 4XN, UK.

## Abstract

The interplay between active biological processes and DNA repair is central to mutagenesis. Here, we show that the ubiquitous process of replication initiation is mutagenic, leaving a specific mutational footprint at thousands of early and efficient replication origins. The observed mutational pattern is consistent with two distinct mechanisms, reflecting the two-step process of origin activation, triggering the formation of DNA breaks at the center of origins and local error-prone DNA synthesis in their immediate vicinity. We demonstrate that these replication initiation–dependent mutational processes exert an influence on phenotypic diversity in humans that is disproportionate to the origins’ genomic size: By increasing mutational loads at gene promoters and splice junctions, the presence of an origin significantly influences both gene expression and mRNA isoform usage. Last, we show that mutagenesis at origins not only drives the evolution of origin sequences but also contributes to sculpting regulatory domains of the human genome.

## Teaser

Activation of DNA replication origins triggers DNA breaks and mutagenesis driving phenotypic diversity in human populations.

## INTRODUCTION

Mutations are the building blocks of genome evolution and the driving force for both Mendelian disorders and cancer. Detailed catalogs of human genetic variation in normal cells and tumors have begun to reveal some of the determinants of mutation distribution in human cells. Large-scale genomic features, including replication timing (*1*) and chromatin structure (*2*), influence the rate of germline and somatic mutations at the megabase scale. Mutation rates are also influenced by local features such as nucleosome positioning (*3*), the orientation of the DNA minor groove around nucleosomes (*4*), transcription factor (TF) binding (*5*, *6*), and transcription initiation (*7*). These analyses, and others (*8*), have established a principle that differential DNA accessibility, due to DNA condensation or active biological processes, significantly contributes to variable local mutation rates in the human genome. DNA replication initiation involves several events with the potential for local DNA damage and mutagenesis, including activation of the replicative helicases and origin DNA melting (*9*), and a series of polymerase switches (*10*). This suggests that origins have the potential to be hotspots for mutagenesis and that local mutation rates should reflect the usage and efficiency of individual origins. However, the lack of high-resolution methods for mapping replication origins and quantifying their efficiency has hampered the characterization of the nature and consequences of the mutational processes operating at replication origins. Here, we take advantage of recent qualitative and quantitative high-resolution maps of human replication origins, generated by others and us, to quantify mutation rates at origins and assess the mutagenic properties of replication initiation.

## RESULTS

### Germline mutation rate is increased at constitutive origins

**Fig. 1.**
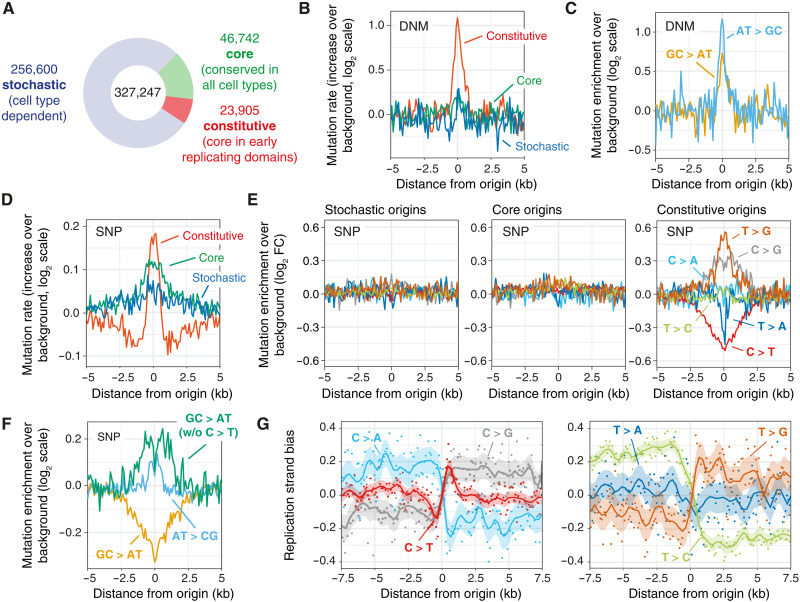
Elevated mutation rates at constitutive origins. (**A**) Categorization of human replication origins based on usage. Stochastic origins (*11*) are cell type–specific, while core (*11*) and constitutive (*12*) origins are replication initiation hotspots independent of cell type. (**B**) DNM rates are increased at constitutive origins compared to their flanking domains [*P* = 4.00 × 10^−4^ (constitutive), 0.44 (core), and 0.87 (stochastic), χ^2^ test]. Mutation rates reported as the increase over background values in domains adjacent to origins. (**C**) Increased mutation rates at constitutive origins are not due to local sequence composition. Correcting for base composition shows an excess of A or T to C or G (AT > CG) and G or C to A or T (GC > AT) variants. (**D**) Population SNPs are increased at all origin types compared to their flanking domains [*P* = 2.46 × 10^−4^ (constitutive), 3.50 × 10^−8^ (core), and 0.014 (stochastic), χ^2^ test]. (**E**) Correcting mutation rates shows that increased SNP mutation rates at stochastic and core, but not constitutive origins, are a consequence of local sequence composition effects. The mutated base is represented by the pyrimidine of the base pair. (**F**) An excess of AT > CG and a reduction of GC > AT polymorphisms is observed at constitutive origins. (**G**) SNPs at constitutive origins display replication strand biases supporting that mutagenesis at constitutive origins is replication dependent. Replication strand bias is computed as the ratio between the density of mutation at a given base pair over the density of mutation at the complementary base pair. As the presence of replication origin clusters may bias mutation rate analyses, we considered only isolated origins (9351 constitutive, 12,224 core, and 10,923 stochastic origins; see Materials and Methods).

To determine the impact of replication initiation on mutation rates, we first categorized human replication origins according to their usage and efficiency. A map of origins, obtained using short nascent strand isolation coupled with next-generation sequencing (SNS-seq), from 19 human cell samples (*11*) representing untransformed and immortalized cell types allowed determination of origin usage. This approach identified 320,748 origins, of which 256,600 were found to be cell type dependent, hereafter referred to as stochastic origins, and 64,148 were found in all cell lines analyzed (Fig. 1A). We refined this dataset by identifying and annotating the subset of cell type–independent origins enriched in early replicating domains of the human genome using our own high-resolution origin mapping technique, initiation site sequencing 2 (ini-seq 2) (*12*), that, as well as providing high-resolution origin position information, gives a quantitative estimate of initiation efficiency that reflects the probability that an origin fires at each cell division. Ini-seq 2 identified 23,905 origins, at a ~1-kb resolution, which are enriched in conserved early replication domains of the human genome (fig. S1A) and significantly overlap (72.8%) with the set of cell type–independent origins. We hereafter refer to the set of cell type–independent origins, identified by SNS-seq, that do not overlap with the ini-seq 2 origins as core origins to follow the nomenclature introduced in (*11*). We subsequently refer to the set of ini-seq 2 origins as constitutive origins as they define replication initiation zones conserved within cell types (*12*) and likely represent the subset of the most active and utilized sites of replication initiation. All three classes of origin are thus distinct and nonoverlapping. As our ini-seq 2 method allows categorization of human replication origins by efficiency, we could demonstrate that origin usage and efficiency are related to origin base composition (fig. S1, B and C).

We first quantified mutation rates by assessing the distribution of single-nucleotide variants from de novo mutations (DNMs) in the vicinity of the three replication origin classes. Because DNMs occur within one generation, i.e., are present in an offspring but not in either parent, and are subject to minimal selective pressure, their distribution reflects the germline mutation rate. We observed an increased frequency of DNMs at constitutive origins, but not at stochastic and core origins, compared with their flanking regions (Fig. 1B). We determined that this elevated mutation rate could not be explained by the sequence context (Fig. 1C), indicating that mutagenesis is increased at early and highly efficient origins. We also assessed the distribution of common single-nucleotide polymorphisms (SNPs) and small insertions/deletions (indels) that arise over hundreds or thousands of generations and reflect both active mutational processes and selective pressures. We found increased SNP frequency and decreased distances between SNPs at all origin types compared with their flanking regions (Fig. 1D and fig. S1D). Mutation frequency for long [≥2 base pairs (bp)], but not short (=1 bp), indels was increased at core and constitutive origins (fig. S1E). When correcting for local variation in base composition, we found that the apparently elevated SNP mutation rate observed at stochastic and core origins is explained by the sequence context (Fig. 1E). However, for constitutive origins, the observed excess of T > G, C > G, T > C, and, to some extent, C > A mutations was not accounted for by local changes in base composition (Fig. 1E). It is notable that C > T transitions, the most frequent mutation in human populations (*13*), are depleted at constitutive origins. Overall, when discounting C > T transitions, we observed an excess of both GC > AT and AT > GC mutations at constitutive origins (Fig. 1F). The four enriched pyrimidine mutation types each exhibited significant strand biases (Fig. 1G), the polarity of which inverts at the origins, supporting a role for DNA replication in their generation. Further, the magnitude of the strand bias was dependent on the efficiency of the constitutive origins (fig. S1F). This replication strand bias was not observed at stochastic and core origins (fig. S1G). These observations suggest that a specific form of replication-dependent mutagenesis is focused on constitutive origins.

### Two distinct mutational processes operate at constitutive origins

**Fig. 2.**
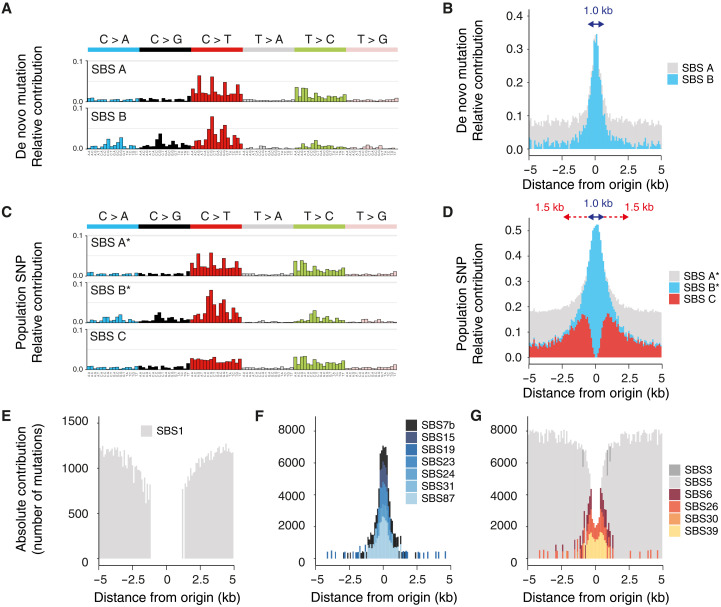
Distinct mutational processes operating at constitutive origins. (**A**) Unbiased de novo mutational signature analysis, computed from the distribution of DNMs, around origins. Each profile shows the mutation probability of each indicated context-dependent base substitution type. (**B**) Contribution of SBS A and B to mutagenesis at origins. Signature exposures are reported as the relative contribution of each signature in 100-bp windows around constitutive origins. (**C**) Mutational signature analysis, computed from the distribution of SNPs, around origins. SNP SBS A* and SBS B* are highly similar to DNM SBS A and SBS B (0.99 and 0.98 cosine similarity, respectively) and hence describe the same mutational processes. (**D**) Contribution of SBS A*, B*, and C to mutagenesis at origins. (**E** to **G**) Comparison of SBS A*, B*, and C to known signatures of somatic mutations in noncancerous and cancerous human tissues. (E) The origin-associated signature SBS A* is mainly explained by the known signature SBS 1, a clock-like background mutational process. (F) SBS B* is reconstructed from a set of seven known signatures associated with DNA damage. (G) SBS C is reconstructed from a set of six known signatures including SBS 5, describing another clock-like background mutational process, and signatures linked with defective MMR.

To characterize the mutational processes associated with origins, we identified the mutational signatures operating at constitutive origins using an established nonnegative matrix factorization (NMF) approach (*14*). We first extracted single-base substitution signatures (SBS) from DNM substitution count matrices reporting the observed frequencies of the six pyrimidine base substitutions in their trinucleotide context. We identified two signatures, which we termed SBS A and B, operating in the vicinity of constitutive origins (Fig. 2A) and analyzed their relative contribution to mutagenesis at these origins (Fig. 2B). While SBS A mainly operates outside constitutive origins, SBS B contributes to mutagenesis within a narrow ~1-kb window centered on the origins. When extracting mutational signatures from SNP mutation count matrices, we identified three signatures, SBS A*, B*, and C (Fig. 2C). SBS A* and B* have similar profiles to SBS A and B (cosine similarity of 0.99 and 0.98, respectively) and thus describe the same mutational processes. SBS C displays a “volcano-shaped” profile extending the footprint of SBS B* by ~1.5 kb either side of the origins with a strong depletion exactly at their center (Fig. 2D). Hence, SBS B and C contribute to mutagenesis within a ~4-kb domain centered on constitutive origins. The contribution of SBS B and C to mutagenesis at origins correlates with constitutive origin efficiency (fig. S2, A and B) and reflects origin usage (fig. S2C), supporting that they are promoted by replication initiation. Signatures SBS B* and C, but not SBS A, exhibit significant strand biases (fig. S2D). The absence of SBS C when considering only DNMs suggests that the mutational process described by this signature induces rarer variants that are observable over background only when accumulated over several generations.

We next identified three small common indel signatures, hereafter referred to ID A, B, and C, operating in concert with SBS A, B, and C, respectively (fig. S2, E and F). We found that ID A and C signatures are mainly composed of indels at long (≥5 bp) homopolymers, indicative of DNA polymerase slippage and incomplete DNA mismatch repair (MMR) (*15*). In contrast, the ID B signature is characterized by increased long (≥5 bp) deletions with at least 5 bp of microhomology at their boundaries, indicative of DNA double-strand breaks (DSBs) processed by alternative end-joining mechanisms (*16*), or microhomology-mediated break-induced replication (MMBIR) (*17*).

To shed light on the molecular mechanisms underlying mutagenesis at constitutive origins, we compared the extracted SBS A-C signatures to known signatures of somatic mutations in noncancerous and cancerous human tissues (*18*). We identified a set of 14 known signatures that most closely reconstruct the SNP mutation profiles of SBS A to C (fig. S3A) and assigned each of these signatures to one of the origin-associated signatures based on their cosine similarity and exposure. Our SBS A signature is mainly explained by the known SBS 1 signature (Fig. 2E), caused by the spontaneous or enzymatic deamination of 5-methylcytosine, and described as a clock-like mutational process that leads to the accumulation of mutations in all cell types in a time-dependent manner (*19*). Hence, SBS A can be considered as a background mutational process. A set of seven signatures, comprising SBS 7b (ultraviolet exposure), SBS 24 (aflatoxin exposure), and SBS 31 (platinum chemotherapy treatment), allows reconstruction of the SBS B profile operating at constitutive origins (Fig. 2F). This set of signatures describes mutagenic processes associated with bulky DNA damage that can lead to replication fork stalling, collapse, and DNA breaks (*20*). Last, a set of six signatures reconstructs the mutagenic processes operating in the flanks of constitutive origins, displaying the volcano-shaped profile of SBS C (Fig. 2G). This set of signatures includes SBS 5, another background clock-like process (*19*), and SBS 6 and SBS 26, two signatures associated with defective MMR (*18*). Exposure of origins to the mutational processes defined by these SBS signatures is replication dependent, as their contribution to origin mutagenesis is usage and efficiency dependent (fig. S3, B and C). Together, these observations suggest that SBS B and C are likely to originate from unrepaired DNA breaks and persistent mismatches induced by replication initiation.

### A model for mutagenesis at origins

**Fig. 3.**
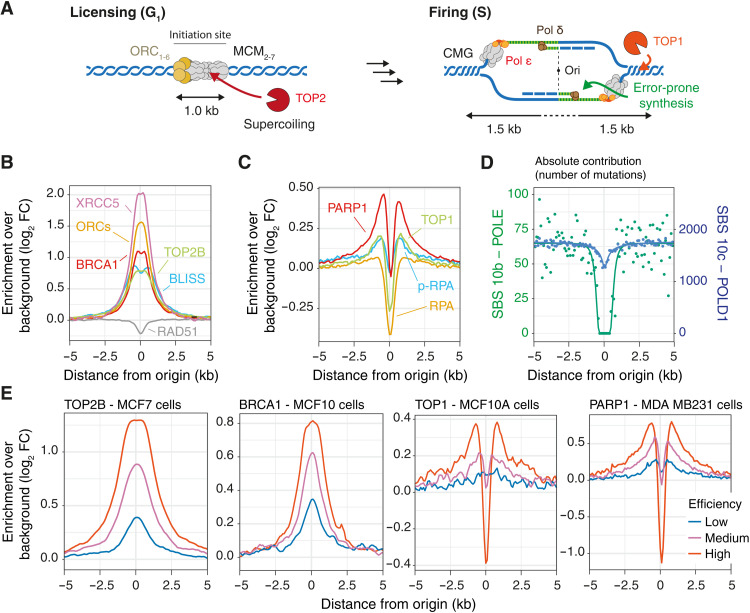
Mutagenesis at origins reflects the two-step process of replication initiation. (**A**) Model for mutagenesis at constitutive origins depicting putative sources for mutagenesis during origin licensing (G_1_ phase) and firing (S phase). Key steps of the model were validated by probing for the presence of DNA damage and repair factors using available ChIP-seq or genomic datasets at the center (**B**) or the flanks (**C**) of constitutive origins. Reported signals are averages from experiments conducted in different cell types when available. (**D**) Absolute contribution of signature SBS 10b and SBS 10c, associated with the activity of POLE and POLD1, respectively. Both signatures are attributed to defective proofreading due to acquired mutations in the exonuclease domains of the polymerases. The pattern of substitutions is consistent with polymerase δ being the main replicative polymerase active in the first 2 kb around origins. The number of mutations attributed to both signatures was computed in 100-bp windows spanning the 20-kb origin domains, and profiles were fitted using sigmoidal functions. (**E**) Efficiency-dependent enrichment of TOP2B, BRCA1, TOP1, and PARP1 at constitutive origins. Coverage plots were computed considering constitutive origins categorized as low-, medium-, and high-efficient origins. Enrichment was computed in 100-bp windows and normalized using background values from domains adjacent to origins for low-, medium-, and high-efficiency origins.

These results lead us to propose a model, summarized in Fig. 3A, to explain the mutagenesis observed at constitutive origins. We validated the principal features of our model by analyzing available chromatin immunoprecipitation sequencing (ChIP-seq) datasets phasing the signals for key proteins on constitutive origins (Fig. 3, B and C) and assessing the impact of origin efficiency on protein enrichment (Fig. 3E).

During the G_1_ phase of the cell cycle, licensing of all potential replication origins starts with the binding of the origin recognition complex (ORC), which displays high affinity for negatively supercoiled DNA (*21*) and binds along with DNA topoisomerase II (*22*, *23*). Consistent with this, we found that ORC proteins colocalize with TOP2B at constitutive origins to define ~1-kb replication initiation sites (Fig. 3B). Abortive topoisomerase II activity at origins leads to potentially mutagenic DSBs (*24*) confirmed by a local increase of BLISS (breaks labeling in situ and sequencing) (*25*) signals at origins (Fig. 3B). The formation of DSBs at origins is further supported by the colocalization of the DNA end binding factor XRCC5/Ku80 (*26*) with origins (Fig. 3B). BRCA1, which physically and functionally interacts with Ku80 to promote classical nonhomologous end joining (NHEJ) (*27*), is also enriched at origins, but the key homologous recombination factor RAD51 is depleted (Fig. 3B). The preferential use of mutagenic end-joining pathways at these breaks is further supported by the ID B signature (fig. S2E), which is characterized by deletions with microhomology consistent with alternative NHEJ (fig. S2D), a pathway known to act redundantly with classical NHEJ (*28*).

At the onset of the S phase, MCM2 to MCM7, which are loaded at origins during the G_1_ phase, are activated, and the two replicative Cdc45-Mcm2-7-GINS helicase complexes translocate in opposite directions. This creates positive torsional constraints in front of the newly activated replication forks, requiring topoisomerase I to ensure continued progression (*29*). Abortive topoisomerase I activity would lead to the accumulation of potentially mutagenic single-stranded DNA breaks (SSBs) in sequences flanking the origins. We found that TOP1 colocalizes with the SSB-sensing factor poly(adenosine diphosphate–ribose) polymerase 1 (PARP1) at the boundaries of the origins (Fig. 3C), but both factors are depleted at the origins themselves. It has been demonstrated in yeast that, after priming of DNA replication by polymerase α, polymerase δ initiates both leading and lagging strand synthesis and thus performs the bulk of DNA synthesis in the vicinity of the origin before handing over to polymerase ε for ongoing leading strand synthesis (*30*–*32*). We thus assessed the exposure of constitutive origins to mutational signatures associated with the activity of POLD1 and POLE, which are enhanced when the exonuclease domains of these polymerases are disrupted (*33*). We found that the substitution pattern is consistent with polymerase δ being the active polymerase within 2-kb domains centered on human origins (Fig. 3D). Further, the accumulation of S33 phosphorylated RPA2 over the background RPA signal in the flanks of the origins suggests local ATR activation indicative of poorly processive and uncoupled DNA synthesis (*34*, *35*) (Fig. 3C). Together, this may lead to an increased burden of mismatches and explain the MMR signatures detected in the origin flanks (Fig. 2G). We found that DNA repair factors and DSBs bind and occur concomitantly at origins (fig. S3D), and that the intensity of all analyzed ChIP-seq signals correlates with origin efficiency (Fig. 3E), supporting that recruitment of DNA repair factors and the accumulation of mutations at origins are due to their activation. Moreover, the volcano-shaped enrichment pattern, associated with PARP1, TOP1, and pRPA at origins, cannot be explained by specific genome features (fig. S3E). Hence, the discrete replication initiation sites at constitutive origins represent hotspots for mutagenesis that have the potential to affect genome evolution and expression.

### Functional consequences of origin activation

**Fig. 4.**
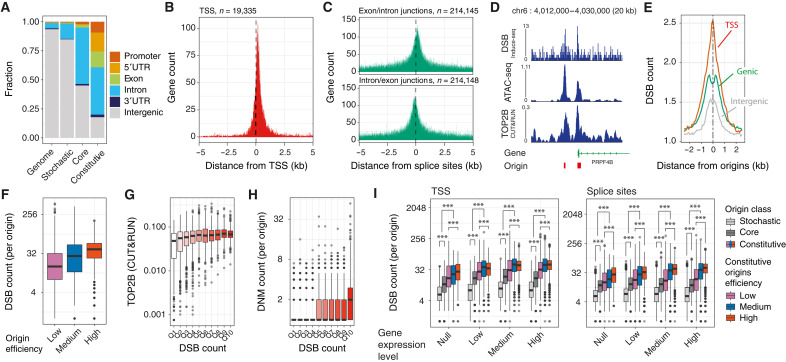
Functional consequences of origin activation. (**A**) Distribution of stochastic, core, and constitutive origins within gene features considering origin midpoints. (**B** and **C**) Distribution of origin midpoints at element junctions, TSSs (B), exon/intron junctions (C), and intron/exon junctions (D). Only protein-coding genes (*n* = 19,335) and GENCODE basic transcripts (*n* = 59,563) were considered to prioritize full-length coding transcripts over partial or noncoding transcripts. (**D**) Representative sequencing tracks showing the accumulation of DSBs, increased DNA accessibility, and enrichment of TOP2B at constitutive origins (red boxes) found at proximity and at the TSS of the PRPF4B gene. Further examples in fig. S4A. Sequencing data are from INDUCE-seq, ATAC-seq, and TOP2B CUT&RUN assays in H9 human embryonic stem cells. (**E**) Distribution of DSBs at origins. DSB counts were computed in windows of 100-nt around ±2.5 kb of origin midpoints. (**F**) Correlation of DSB counts with origin efficiency at constitutive origins (*P* < 2.2 × 10^−16^, χ^2^ test). (**G**) Local TOP2B signal intensity at intergenic origins (*P* = 8.12 × 10^−3^, χ^2^ test) as a function of DSB counts in 10 quantile bins. (**H**) DNM counts mapped at intergenic origins as a function of DSB counts in 10 quantile bins (*P* < 2.2 × 10^−16^, χ^2^ test). (**I**) Impact of origin usage, efficiency, and expression levels of associated genes on DSB density in H9 cells at origins within 2.5 kb of either TSSs (left) or splice sites (right). *P* values for the comparison of the distribution of individual values were calculated using Kolmogorov-Smirnov tests, ***P < 0.001.

To assess the functional consequences of origin activation, we first analyzed the distribution of origins within the human genome (Fig. 4A). We found that all origin classes are enriched within genes (*P* < 2.2 × 10^−16^ for all origin classes, χ^2^ test), with ~80% of constitutive origins lying within gene bodies. Constitutive origins locally accumulate at gene element boundaries such as transcription start sites (TSSs) and splice junctions (Fig. 4, B and C), suggesting that replication initiation may locally affect the integrity of protein coding genes. We thus aimed to assess the local enrichment of DSBs at constitutive origins as predicted by our model discussed above. We selected the H9 human embryonic stem cell line, in which both core and constitutive origins are active, and used a combination of DSB mapping, ATAC-seq (Assay for Transposase-Accessible Chromatin using sequencing), and CUT&RUN assays to map chromatin accessibility and topoisomerase distribution to validate our model (Fig. 3A). To map DSBs, we used INDUCE-seq, which allows the detection of low-level DSBs caused by physiological processes and provides a per-base absolute count of DSB that is not distorted by polymerase chain reaction (PCR) amplification bias (*36*). We found a local accumulation of DSB and an increase in ATAC-seq and TOP2A/B signals at both genic and intergenic constitutive origins (Fig. 4D and fig. S4A). INDUCE-seq analysis revealed higher DSB densities, compared to the background level, at constitutive origins independently of their genomic location, with a higher level for origins at gene TSSs or within gene bodies (Fig. 4E). However, DSB densities at all origins (Fig. 4F) and, separately, intergenic origins (fig. S4B) correlate with origin efficiency (*P* < 2.2 × 10^−16^ and *P* = 2.71 × 10^−11^, respectively, χ^2^ tests of independence). This suggests that origin activation, rather than origin environment, is the cause of DSB accumulation. We observed the same trends when considering the ATAC-seq signal (fig. S4, C and D) and found that the number of DSBs at all origins or intergenic origins correlates with TOP2B (Fig. 4G and fig. S4F) and TOP2A (fig. S4G) enrichment (*P* from 7.46 × 10^−3^ to 2.64 × 10^−2^ for all tested conditions, χ^2^ test of independence). These observations suggest that the sites of efficient replication initiation in accessible chromatin are associated with accumulation of topoisomerases and are hotspots for DSBs. Further, the number of DSBs detected at constitutive origins correlates with the number of DNMs detected in human populations at the same sites (*P* < 2.2 × 10^−16^, χ^2^ test of independence; Fig. 4H), suggesting that the accumulation of DSBs at origins in the H9 cell line is a good proxy for mutagenesis in the germline.

Because constitutive origins are enriched within gene bodies (Fig. 4A), we assessed the contribution of transcription to the accumulation of DSBs at origins. We estimated gene expression levels from RNA sequencing (RNA-seq) of the H9 cell line and assessed the contribution of replication initiation and transcription on DSB densities at gene TSSs and splice sites. We found that both TSSs and splice sites marked by constitutive origins display higher density of DSB than those that do not contain origins (*P* = 1.95 × 10^−5^ and 3.72 × 10^−12^, respectively, Kolmogorov-Smirnov tests; fig. S4H). However, we also found that the density of DSBs at TSSs and splice sites depends on the expression of the associated genes (fig. S4I). To further understand the relationship between origins and transcription in generating DSBs at origins, we assessed the impact of origin usage, efficiency, and expression levels of the associated genes on DSB density at origins found at either TSSs or splice sites (Fig. 4I). We found that the number of DSBs at origins correlates with origin usage and efficiency (*P* < 2.2 × 10^−16^ for both tests, χ^2^ tests of independence) independently of gene expression, meaning that the contribution of transcription to DSB density at origins is minimal. When comparing origins at TSSs or splice sites of genes expressed at similar levels, increased origin usage/efficiency (i.e., comparing constitutive against stochastic origins) leads to a ~5.2-fold increase in DSBs, while increased expression (i.e., origins at sites of nonexpressed against highly expressed genes) leads to a ~1.9-fold increase in DSBs. These observations show that the presence of efficient origins triggers a local accumulation of DSB independently from transcription. We note that the presence of constitutive origins in a gene does not impede its overall transcriptional activity (fig. S4J).

### Constitutive origins drive phenotypic diversity

**Fig. 5.**
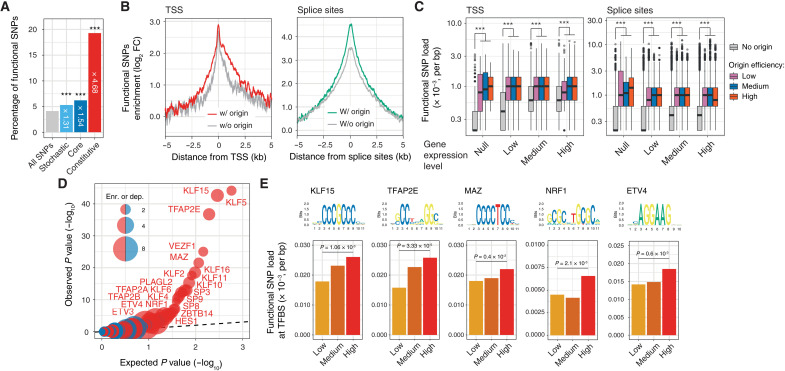
Constitutive origins increase mutational burden in their host genes. (**A**) Percentages of variants within 5-kb domains centered on origins predicted to have regulatory potential. (**B**) The presence of constitutive origins increases functional variants at gene TSSs (left) and splice sites (right). Protein coding genes categorized by the presence (*n* = 11,410) or absence (*n* = 7925) of constitutive origins within 2.5 kb of their TSS. Splice sites of GENCODE basic transcripts categorized according to the presence (*n* = 55,903) or absence (*n* = 121,511) of constitutive origins within 2.5 kb. Mutation densities presented in bins of 100 bp and normalized using background values from adjacent domains. (**C**) Impact of origin efficiency and expression levels of associated genes on functional SNP load at TSSs (left) or splice sites (right). Expression levels reflect gene expression in adult male germ cells. ***P < 0.001, Kolmogorov-Smirnov test. (**D**) Mutagenesis at constitutive origins disrupts TF binding. Quantile-quantile plot reporting the association of origin variants with TF binding sites (TFBSs). *P* values computed 2 × 2 contingency χ^2^ tests and compared to the normal expected uniform distribution to identify TFBS disrupted by constitutive origin-associated functional variants. Enrichment and depletion values are reported by the size of red and blue bubbles, respectively. (**E**) Functional SNPs at constitutive origins overlap with specific GC-rich TFBSs. Mutational loads at transcription factor (TF) binding sites associated with constitutive origin functional variants correlate with origin efficiency. *P* values calculated using χ^2^ tests of independence.

The enrichment of constitutive origins at element boundaries (Fig. 4, B and C) suggests that variants associated with constitutive origins are more likely to have a functional impact. To assess whether this is the case, we used RegulomeDB (*37*) and found a significant enrichment at constitutive origins of SNPs with predicted effects on gene regulation, hereafter referred to as functional variants (RegulomeDB score ≤ 2; a 4.68-fold increase when compared to all SNPs; Fig. 5A). This enrichment reflects replication initiation as it increases with origin usage (Fig. 5A) and correlates with constitutive origin efficiency (*P* = 7.49 × 10^−206^, χ^2^ test of independence; fig. S5A). We then investigated whether the presence of a constitutive origin within a gene feature increases the mutational load in the associated gene. We first categorized genes according to the presence or absence of a constitutive origin at their TSSs or at splicing sites and computed the density of functional variants around their TSSs and splice sites (Fig. 5B). We found that gene features marked by an origin display increased mutation load compared with those that are not. Thus, genes with an origin within 2.5 kb of their TSSs display a ~1.55-fold increase in mutational load (Fig. 5B and fig. S5B) compared to those without, and this enrichment correlates with origin efficiency (*P* = 2.72 × 10^−6^, χ^2^ test of independence; fig. S5C). Similarly, splice sites marked by an origin display a ~1.34-fold increase in mutational load (Fig. 5B and fig. S5B) that also corelates with origin efficiency (*P* = 8.3 × 10^−101^, χ^2^ test of independence; fig. S5C). We then investigated how transcription contributes to this observed increase in mutational loads. To do this, we computed mean levels of gene expression in adult male germ cells from the spermatogonia, spermatocyte, and spermatid lineages (*38*) and assessed the impact of origins and transcription on the density of functional SNPs at TSSs and splice sites (Fig. 5C). We found that the presence of an origin significantly increases the mutational load at these sites even at nonexpressed genes and that the presence of an origin leads to a ~1.34- to 2.8-fold increases when considering gene of similar expression levels. Our observations support replication initiation as an important determinant of functional SNP burden within protein coding genes and suggest that mutagenesis at origins has the potential to be translated into gene expression variation and to modulate mRNA splicing.

**Fig. 6.**
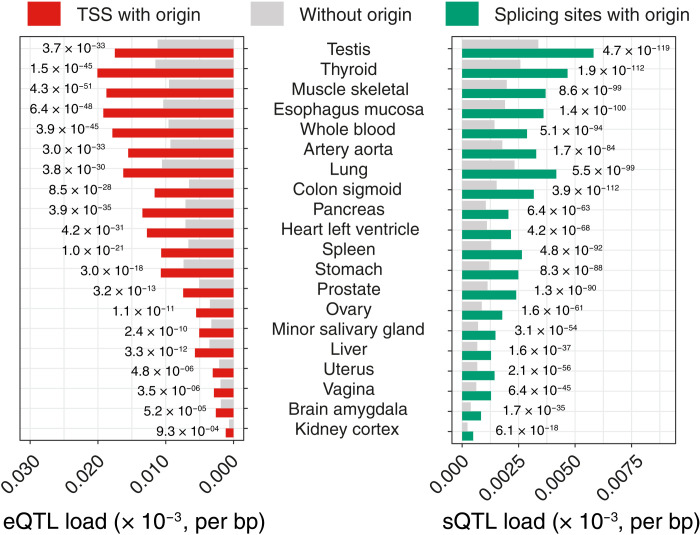
Constitutive origins drive phenotypic diversity in human populations. Cis-eQTLs and cis-sQTLs are enriched at the TSSs and splice sites of genes, respectively, marked by constitutive origins compared to sites free of origins. Cis-eQTL and cis-sQTL densities within 5-kb regions centered on gene features were computed for features marked by constitutive origins (red and green bars for TSSs and splice sites, respectively) or free from origins (gray bars) in 49 human tissues. The figure reports a selection of tissues (the full figures are reported in fig. S6, A and B). *P* values computed using χ^2^ tests.

To test whether this is the case, we first asked whether origin-associated SNPs have the potential to alter TF binding sites (TFBSs). We extracted the sequence context of all functional variants found within 2.5 kb around TSSs and created lists of predicted TFBSs associated with the functional variants. By comparing the enrichment of TF motifs around TSSs marked by an origin compared with those that are origin free (Fig. 5D), we observed enrichment of GC-rich binding sites (Fig. 5E), including those of members of the Sp/KLF and AP-2 families that play a vital role in regulating the growth and development of a large number of tissues (*39*, *40*). Mutational load at these TFBSs correlates with origin efficiency (Fig. 5E), suggesting that mutagenesis at constitutive origins molds TF binding in a replication-dependent manner. We then investigated whether alteration of TF binding by origin mutagenesis induces gene expression variation in human populations. Using datasets generated by the Genotype-Tissue Expression (GTEx) project (*41*) to identify cis-expression quantitative trait loci (cis-eQTL)/gene pairs, we analyzed mutational loads in 2.5-kb regions around TSSs marked by constitutive origins, and those without, and computed cis-eQTL enrichment in both sets. For the 49 screened tissues, we found an increase in cis-eQTL loads, ranging from 1.70- to 2.87-fold (*P* < 2.7 × 10^−3^, χ^2^ test; Fig. 6 and fig. S6A), in gene TSSs marked by an origin. A similar analysis revealed that splice sites marked by an origin display increased cis-splicing quantitative trait loci (cis-sQTL) loads (up to 2.15-fold with *P* < 6.1 × 10^−18^, χ^2^ test; Fig. 6 and fig. S6B), supporting the notion that mutagenesis at constitutive origins also affects mRNA splicing. Together, these results suggest that genetic variants induced by mutagenesis at constitutive origins are a source of gene expression variation and phenotypic diversity in human populations.

### Mutagenesis drives the evolution of origin sequences

**Fig. 7.**
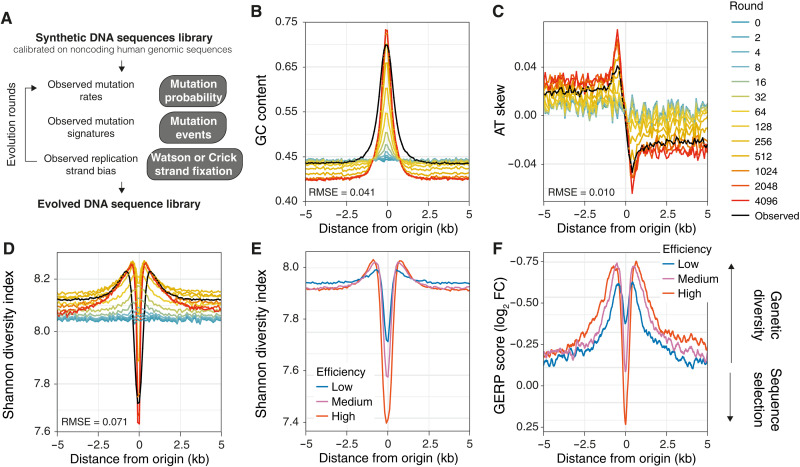
Mutagenesis at origins drives origin specification and genetic diversity. (**A**) An in silico model was devised to assess the impact of mutagenesis during origin activation on the evolution of sequences at constitutive origins. The model uses rules (see Materials and Methods) defined on observed mutation rates, mutagenic processes, and replication asymmetry at constitutive origins to evolve a library of 1000 DNA sequences calibrated on noncoding regions of the human genome. Applying sequential rounds of evolution recapitulates features of constitutive origins such as (**B**) local increase in GC content, (**C**) AT skew, and (**D**) sequence diversity computed using Shannon diversity indexes. Colored lines represent values at different rounds of evolution, and black lines correspond to values observed at constitutive origins. The root mean square error (RMSE) values are the SDs of the residuals between computed values at round 4096 and observed values. (**E**) Shannon diversity indexes decrease, indicating sequence selection, and increase, indicating sequence diversity, at origin centers and flanks, respectively. This is replication dependent as the signal depends on the efficiency of constitutive origins. (**F**) Rates of genome evolution, plotted as GERP scores, at constitutive origins are replication dependent and display positive and negative values at the centers and flanks of origins, respectively. GERP scores were normalized using background values from domains adjacent to origins. Positive and negative GERP scores indicate an increase and decrease in nucleotide substitution rates relative to a genome-wide expectation of neutral evolution, respectively. For (E) and (F), origins were binned according to low (blue), medium (purple), or high (red) efficiency.

To investigate the long-term consequences of mutagenesis at constitutive origins on the evolution of the human genome, we devised an in silico model in which a library of DNA sequences, calibrated on noncoding human genomic sequences, was evolved according to rules defined by the observed mutation prevalence, pattern, and replication strand bias at constitutive origins (Fig. 7A). We computed probability density functions (PDFs; fig. S7, A to C) that describe the probability that a nucleotide will be mutated based on its position relative to the origin and the nature of that mutation based on the trinucleotide context and exposure to the known SBS signatures, corrected for base composition. We found that our model recapitulates general features of origins observed in the human genome such as local increase of GC content (Fig. 7B), base composition skews (Fig. 7C and fig. S7D), and the presence of origin cis-regulatory elements such as CpG islands (*42*) and G-quadruplexes (*43*) (fig. S7, E and F). We note that the strand biases associated with signature SBS B* and C (fig. S2D) allow us to explain the specific profiles of the base composition skews. This observation supports the hypothesis that the mutagenic processes operational at origins drive origin sequence evolution toward that observed in the genome of modern humans.

We next analyzed variation in the Shannon diversity index, a measure of sequence diversity, around our modeled in silico origins (Fig. 7D). Sequence diversity decreases over time at origins but increases in their flanks. The resulting volcano-shaped patterns around origins, reminiscent of the exposure distribution of signatures SBS B and C (Fig. 2D), suggest that the mutational processes linked to replication initiation have the capacity not only to create the sequence environment observed at replication origins but also to drive genetic diversity in the immediate vicinity of origins. These impacts are dependent on origin efficiency (*P* = 8.26 × 10^−4^, χ^2^ test of independence; Fig. 7E), suggesting that the most efficient sites of replication initiation contribute to the evolution of the human genome. To test this hypothesis, we analyzed the rate of genome evolution around constitutive origins by assessing local variation of the genomic evolutionary rate profiling (GERP) score, which quantifies nucleotide substitution rates relative to a genome-wide expectation of neutral evolution (*44*). At origin centers, we found positive GERP scores, indicating purifying selection for functional elements, while in the flanks, we observed negative GERP scores, indicating increased nucleotide substitution rates (Fig. 7F). Furthermore, increases in substitution rate correlated with origin efficiency (*P* < 2.2 × 10^−16^, χ^2^ test of independence; Fig. 7F) and usage (fig. S7G), demonstrating that genetic diversity is linked to the efficiency of replication initiation. Our results show that the presence of an origin imposes local evolutionary constraints and, because of their specific genomic location (Fig. 4, A to C), contributes to the evolution of transcriptional units.

## DISCUSSION

Here, we establish that a class of highly efficient replication origins allows the observation of specific mutational patterns associated with human replication initiation that substantially affect genome evolution and expression. The mutational processes we report are distinct, by both their nature and consequences, from the known processes that have been previously associated with replication (*8*). Described replication-associated mutagenic processes have been linked to ongoing replication forks or transcription/replication conflicts, leading to DNA damage and mutations that are distributed along specific domains of the human genome. Recent analysis of the distribution of human germline mutations (*45*) have uncovered three independent mutational processes associated with ongoing replication that result from the strand-specific resolution of bulky DNA damage and replication errors. These processes are operating within either ~100-kb gene-rich domains of the genome, correlating with gene expression, or ~500-kb gene-poor late replicating domains. In contrast, the replication initiation–associated mutagenesis we describe here is focused to ~1-kb loci defined by early and efficient origins in a transcription-independent manner. It is also worth noting that the mutagenic processes associated with replication elongation are dominated by T > C, C > T, and C > A mutations (*45*, *46*) and that the probability of these substitutions occurring in the immediate vicinity of constitutive origins is not increased when corrected for base composition (Fig. 1E). Our data support a model in which mutagenesis at origins is linked to the formation of DNA breaks at the center of the origin followed by an initial phase of error-prone synthesis as the forks escape the origins, likely due to the use of polymerase δ on both leading and lagging strand before the leading strand switches to a fully processive mode using polymerase ε (*30*, *31*). We demonstrate that TOP2 accumulates at constitutive origins and hypothesize that breaks at origins originate from abortive TOP2 activity, but it is worth noting that origin firing requires RNA primers whose removal may also lead to persistent breaks that could contribute to the observed increase in break levels at origins.

The accumulation of DNMs at constitutive origins provides direct evidence for increased germline mutation rates at early and efficient origins, supporting the mutagenic properties of origin activation. However, we note that stochastic and core origins exhibit a similar mutation rate to their flanking regions (Fig. 1B). This could be due to the lower burden of origin mutagenesis at these less frequently used sites of replication initiation that is not separable from the background, with only the most recurrently used origins accumulating a significant mutational imprint. Moreover, the inherently low rate of germline mutation may hamper the detection of low-frequency variants at less efficient origins. We found elevated mutation rates at all origin classes when considering population SNPs (Fig. 1D), suggesting that low-frequency variants accumulate at origins over generations. However, increased SNP mutation rates at stochastic and core origins are explained by the local variation of base composition, indicating that no specific mutational processes can be detected at these sites and that no evolutionary forces contribute to their fixation. This point is further supported by the fact that these origins experienced a rapid turnover during vertebrate evolution (*47*).

A direct relationship between constitutive origin activation and mutagenesis is supported by the significant correlations between origin efficiency and mutation frequency. We identified two replication-dependent mutational processes operating at constitutive origins that may result from the two-step process of origin activation (Fig. 3A). SBS B is detected when considering both DNMs and SNPs, but SBS C is revealed only when considering SNPs (Fig. 2, B and D). This suggests that while the mutational process associated with SBS B induces frequent and persistent mutations, most likely due to the highly mutagenic properties of DSBs, SBS C induces rarer variants that are not at first observed because of the limited number of mapped DNMs. Accumulation over several generations allows observation of these SBS C–associated mutations. It may also suggest that sequences flanking constitutive origin domains are under strong selective pressure that allows the fixation of these mutations in the human population.

Using the H9 experimental cell system, we confirmed the mutagenic properties of constitutive origins by demonstrating the impact of origin activation on genetic instability. We found that, while transcription contributes to the accumulation of DSBs at TSSs and splice sites, origin efficiency is the main determinant of DSB accumulation at origins (Fig. 4I). It is noteworthy that intergenic origins display elevated densities of DSBs compared to their flanking sequences (Fig. 4E) that correlate with origin efficiency (fig. S4B) and TOP2A/B activity (Fig. 4G and fig. S4G). We can then confidently exclude the role of transcription or other mutational processes (*8*) acting at or adjacent to replication origins for explaining the observed mutation landscape of constitutive origins.

We demonstrated that origin usage and activation is the driving force for origin sequence evolution and impose the positioning of local nucleotide skews (Fig. 7C and fig. S7D) known as S-jumps. S-jumps are found at bacterial, viral, and eukaryotic origins (*48*, *49*), suggesting that the role of origins in shaping genomes is conserved throughout evolution. We note that while constitutive origins cover only ~0.7% of the human genome, their enrichment within gene bodies leads to a high local mutation density with disproportionate functional consequence. From an evolutionary perspective, it may seem paradoxical that a replication initiation–coupled mechanism focuses mutation to promoters or splice sites. However, this targeted mutagenesis may provide a significant advantage in shaping the evolution of the multiple promoters (*50*) and alternative splicing sites (*51*) found in most human genes and thus influence the diversity of tissue-specific mRNA isoforms available for selection of new functions.

Although the mutagenesis we report here reflects evolutionary events occurring during normal gametogenesis and early development, we anticipate that dysregulation of mechanisms that maintain genome stability in cancer will modulate mutagenesis at replication initiation sites and exert an additional mutational burden on genetic elements marked by constitutive origins. Knowing that oncogene induction also activates origins within highly transcribed genes (*52*), we anticipate that mutagenesis arising from origin activity may contribute extensively to the evolution of cancer genomes.

## MATERIALS AND METHODS

### Human reference genome and annotation

All analyses were performed on the hg38/GRCh38 human reference genome assembly. Datasets originally obtained with coordinates on other assemblies were projected onto the hg38 assembly using “liftOver” from the rtracklayer R/Bioconductor package (*53*), with the corresponding chain files obtained from http://hgdownload.cse.ucsc.edu in the individual assembly download sections. Gene structure annotations were extracted using the biomaRt R/Bioconductor package (*54*). We considered only TSSs of protein coding genes (*n* = 19,335) and splice sites of GENCODE basic transcripts (*n* = 59,563) to prioritize full-length coding transcripts over partial or noncoding transcripts. Intron coordinates were recovered by subtracting the coordinates of exons, 5′ untranslated regions (5′UTRs), and 3′UTRs from transcript coordinates using the “subtract” command of bedtools (*55*).

### Replication origins and timing

Replication origins, mapped using SNS-seq, from 19 human cell samples were recovered from Akerman *et al.* (*11*). Early replication origins, mapped by ini-seq 2, were recovered from (*12*). Stochastic origins are cell type–dependent origins as identified by SNS-seq. In this article, core origins are the cell type–independent origins identified by SNS-seq that do not overlap with origins mapped with ini-seq 2. We consider that two initiation sites represent the same origin if the mapped domains are found within less than 5 kb. Distances between origins were computed using the “closest” function of bedtools. To demonstrate the effect of origin efficiency on mutagenesis, we defined three groups of constitutive origins, i.e., origins mapped using the ini-seq 2 protocol, termed low-, medium-, and high-efficiency constitutive origins, by dividing the distribution of observed efficiencies into three quantiles and assigning each origin to one of the three groups (*12*). Replication timing was computed using data from Hansen *et al.* (*56*) considering the log_2_ ratio of early-replicating (G_1_ + S_1_) to late-replicating (S_4_ + G_2_) DNA values as a function of genomic positions in 50-kb sliding windows at 1-kb intervals. We averaged the obtained timing values from experiments using the GM12812, BG02, IMR-90, and MCF7 cell lines. Enrichment of replication origins within replication timing domains was assessed by computing the fraction of origins and associated replication timing values in 100-kb windows of the human genome.

### Mutation data

Whole-genome DNMs were obtained from the Gene4Denovo database (*57*). Gene4Denovo integrates 613,193 single-nucleotide variant DNMs across 24 phenotypes from more than 60 publications. The Gene4Denovo database integrates results from trio-based genetic projects and aims to identify variants in offspring’s genomes that are absent in their parents. Hence, DNMs occur in a single generation and can be used to infer germline mutation rates. Whole-genome common population variants were obtained from dbSNP build 151 on hg38. The common subset reports all variants representing alleles observed in the germline with a minor allele frequency of at least 1% and mapped to a single location in the reference genome assembly. Taken as a set, common variants are less likely to be associated with severe genetic diseases due to the effects of natural selection, following the view that deleterious variants are not likely to become common in the population. SNPs (*n* = 34,082,281) and short insertions/deletions (indels, *n* = 3,673,496) were recovered using VCFtools (*58*) and analyzed independently.

### Mutation rate estimation

To compare mutation rates at origins to their neighboring regions, we considered 10-kb flanks either side of origin midpoints. As the presence of replication origin clusters may bias the mutation rate analyses, we considered only domains that contain a single origin. Analyses reported in Figs. 1 and 2 were thus performed considering domains covering 9351 constitutive, 12,224 core, and 10,923 stochastic origins. Local mutation rates were computed, in 100-bp windows covering 10-kb domains centered on origin midpoints, as the ratio of the total number of mutations, DNMs, SNPs, or indels over the total number of considered domains. We corrected the local mutation rates by the basal levels of mutation and considered the increases of mutation rate over background by normalizing the previous values by the average values obtained for the first and last 20 windows of origin domains. Mutation interdistances were computed for each mutation as the smallest distance between the first upstream or downstream mutation, and we averaged values over 100-bp windows covering origin domains. To assess whether mutation rates at origins are due to the local sequence context, we considered the total number of the six pyrimidine mutations, divided by the number of C/G or A/T for C and T mutations, respectively, and considered the enrichment of mutations over background by normalizing the previous values by the average values obtained for the first and last 20 windows of origin domains. This corrects for mononucleotide compositional biases that are abundant in the vicinity of replication initiation sites.

### Replication strand bias

The total number of mutations, *n*, corresponding to a given base pair change *b1:b2* → *m1:m2* and its complementary mutation *b2:b1* → *m2:m1* were computed in 100-bp windows covering the 20-kb origin domains previously defined. Replication strand biases (RSBs) for each window were then calculated as ${\text{RSB}}_{b\to m}={\text{log}}_{2}\;(\;\frac{n_{b1:b2\to m1:m2}}{n_{b2:b1\to m2:m1}}\;)$. Trends in replication strand biases and inversion of asymmetry at constitutive origins are highlighted using local Loess regression fits.

### Mutation signatures analysis

Evaluation and visualization of mutational patterns at origins were performed using the MutationalPatterns (*14*) and NMF (*59*) R/Bioconductor packages. De novo extraction of mutational signatures was performed using an unsupervised NMF approach. We defined mutation count matrices by considering the frequencies of SNPs, calculated in each of the possible 96 trinucleotide 5′ to 3′ contexts, or indels, calculated considering size, nucleotides affected, and presence of repetitive and/or microhomology regions, in 100-bp windows spanning the 20-kb constitutive origin domains defined previously. We determined the number of SBS and indel (ID) signatures by considering the smallest rank at which the cophenetic correlation coefficient starts decreasing. We thus extracted three SBS and ID mutational signatures using 1000 iterations to achieve stability and avoid bad local minima. Signature exposures to not only constitutive, core, and stochastic origin domains but also domains covering low-, medium-, and high-efficiency constitutive origins were constructed by considering the relative contribution of the SBS A to C and ID A to C signatures in 100-bp windows spanning the 20-kb origin domains. To assess the replication dependency of SBS A to C, we considered the relative contribution of each signature to domains around origins where the signatures operate. We considered 1-kb domains, 4-kb domains but omitting the previous domains, and 16-kb domains excluding the previous ones centered at the origins for SBS B, C, and A, respectively.

To determine the underlying molecular mechanisms associated with the extracted SBS A-C signatures, we analyzed the contribution of known signatures of somatic mutations in noncancerous and cancerous human tissues, collected by the Catalogue of Somatic Mutations in Cancer (COSMIC v3.2) and available from https://cancer.sanger.ac.uk/signatures/sbs, to mutagenesis at constitutive origins. To identify the minimum set of mutational processes operating at constitutive origins and avoid signature misattribution, we used an iterative fitting approach with the “fit_to_signatures_strict” function of the MutationalPatterns package and a “cutoff max_delta” of 0.004. We then assigned to each operating signature one of the origin-associated signature SBS A to C based on their cosine similarity and exposure. Known signature exposures to not only constitutive, core, and stochastic origin domains but also domains covering low-, medium-, and high-efficiency constitutive origins were constructed by considering the absolute number of mutations attributed to the operating signatures in 100-bp windows spanning the 20-kb origin domains.

To determine the contribution of polymerase ε and δ to DNA synthesis in the vicinity of constitutive origins, we analyzed the contribution of mutational signatures attributed to defective proofreading due to acquired mutations in the exonuclease domains of POLE or POLD1. To do this, we used an iterative fitting approach, as described previously, but using only the polymerase-associated signatures SBS10a, SBS10b, SBS10c, and SBS10d (COSMIC v3.2) and found that SBS10b (defective POLE proofreading) and SBS10c (defective POLD1 proofreading) contribute to mutagenesis at constitutive origins. Calculating the absolute number of mutations attributed to both signatures in 100-bp windows spanning the 20-kb origin domains allows the demonstration that polymerase δ, but not polymerase ε, is active in a small 4-kb domain centered at the origins.

### DNA repair factor enrichment analysis

The distribution of DNA repair factors and DSBs around constitutive origins was assessed using available ChIP-seq and other datasets. The references and associated Gene Expression Omnibus (GEO) accession numbers for the datasets used in these analyses are reported in table S1. We selected datasets assessing the chromatin distribution of ORC (ORC1 and ORC2 subunits, *n* = 2), TOP2B (*n* = 2), DSBs (BLISS, *n* = 3), BRCA1 (*n* = 4), XRCC5 (*n* = 2), RAD51 (*n* = 1), TOP1 (*n* = 3), PARP1 (*n* = 3), RPA (RPA1 and RPA2 subunits, *n* = 2), and phospho-RPA2-S33 (*n* = 1). We then computed coverage plots using the “map” function of bedtools in windows of 100 bp covering not only 20-kb domains centered on constitutive origins but also domains covering low-, medium-, and high-efficiency constitutive origins. Enrichments over background were computed by normalizing the local enrichment values by the average values obtained for the first and last 20 windows of origin domains. We finally averaged the coverage plots over the different analyzed cell lines and conditions when available.

### Genomic characterization of the H9 experimental cell system

Human H9 embryonic stem cells (WiCell WA09) were cultured at 37°C, 5% CO_2_ in Essential 8 Flex medium on Vitronectin-XF–coated (STEMCELL Technologies) plates. Cells were passaged every 3 to 4 days based on cell density. Cells tested negative for mycoplasma contamination throughout the study. All DNA libraries were quantified by quantitative PCR using the KAPA Library Quantification Kit for Illumina Platforms (Roche) and were tested for quality and primer and adapter contamination using Agilent 2100 Bioanalyzer High Sensitivity DNA chip before proceeding to high-throughput sequencing.

#### Double-stranded DNA break mapping by INDUCE-seq

Cells were harvested using Accutase (Thermo Fisher Scientific) to obtain a single-cell suspension and counted, followed by two washes in Dulbecco’s phosphate-buffered saline (PBS). Cells (~100,000) were deposited into a poly-d-lysine–coated 96-well plate (Greiner Bio-One). Once adhered by centrifugation, cells were cross-linked in 4% paraformaldehyde (Thermo Fisher Scientific) for 10 min followed by two washes with 1× PBS. Plates were tightly sealed, and cells were kept in 1× PBS at 4°C until downstream library preparation. INDUCE-seq was performed as previously described (*36*). Sequencing was performed on Illumina NextSeq 500 using 1 × 75 bp high-capacity flow cell. INDUCE-seq was performed in duplicate. After assessing reproducibility by comparing the genome-wide densities of DSBs in 10-kb windows, replicates were combined.

#### Assay for transposase-accessible chromatin sequencing

ATAC-seq was performed as previously described (*60*) starting from 50,000 fresh nuclei and using the Tn5 Tagment DNA TDE1 Enzyme and Buffer Kit (Illumina). After DNA purification (Qiagen MinElute PCR purification kit), library fragments were amplified using the KAPA HiFi HotStart ReadyMix and 1.25 μM of custom Nextera PCR primers (*61*). Libraries were purified using double (0.5× to 0.7×) SPRI AMPure XP beads (Beckman Coulter) purification. Sequencing was performed on Illumina NovaSeq 6000 using 2 × 150 bp high-capacity flow cells. ATAC-seq was performed in triplicates. After assessing reproducibility by comparing the genome-wide ATAC-seq signal intensity in 10-kb windows, replicates were combined.

#### CUT&RUN assays

TOP2A and TOP2B CUT&RUN assays were performed starting with 100,000 cells using the CUT&RUN Assay Kit (Cell Signaling Technology) and following the manufacturer’s instructions. Primary antibodies were incubated overnight at 4°C [Anti-Topoisomerase II alpha (Abcam, ab52934) and Anti-Topoisomerase II beta (Abcam, ab72334)]. DNA was purified from input and enriched chromatin samples using DNA spin columns (Cell Signaling Technology). CUT&RUN libraries were prepared using the NEBNext Ultra II DNA Library Prep Kit for Illumina following the CUT&RUN assay kit recommendations to avoid denaturation and loss of small DNA fragments. CUT&RUN assays were performed in biological triplicate, and each was sequenced three times to ensure high-quality signals. After assessing reproducibility by comparing the genome-wide CUT&RUN signal intensity in 10 kb windows, replicates were combined.

#### RNA sequencing

RNA was isolated using the RNeasy Kit (Qiagen) following the manufacturer’s instructions and quantified before running on an Agilent RNA Pico 6000 chip using an Agilent 2100 Bioanalyzer. RNA with a RIN (RNA integrity score) score above 7 was used to generate RNA libraries. Libraries were prepared using a NEBNext Ultra II RNA library preparation kit and a NEBNext human rRNA depletion kit (New England Biolabs). Complementary DNA libraries were generated from 750 ng of RNA, amplified for eight PCR cycles, and barcoded with NEBNext Multiplex Oligos for Illumina (New England Biolabs). RNA-seq analysis was performed in triplicate.

#### Data processing

INDUCE-seq reads were processed as previously described (*36*). ATAC-seq, CUT&RUN, and RNA-seq sequence files were quality-trimmed with trim_galore (v0.4.4) (*62*) and aligned to the human genome (GRCh38/hg38). ATAC-seq and CUT&RUN samples were aligned with bowtie2 (v2.2.6) (*63*) using the following parameters for ATACseq (--local) and CUT&RUN (--local --very-sensitive-local --no-unal --no-mixed --no-discordant -I 10 -X 700). Alignments were then filtered with samtools for maximum quality alignments (-q 42), converted to bigwigs (--normalizeUsing BPM --smoothLength 350 --extendReads 150 --centerReads) at binSizes of 50 nucleotides (nt), and normalized to input when relevant. RNA-seq samples were aligned with tophat (v2.1.1) (*64*), and transcript abundances were computed with cufflinks (v2.2.1) (*65*). Gene expression levels were computed by averaging expression values, expressed in transcripts per million (TPM), from the triplicates. Nonexpressed genes are genes with <0.01 TPM values. Low-, medium-, and high-gene expression levels were computed by dividing the distribution of remaining expression values into three quantiles.

### Functional variants annotation and distribution

To test whether origin-associated SNPs are likely to have a functional impact on gene expression, we used RegulomeDB (*37*). RegulomeDB annotates SNPs with known and predicted regulatory elements within the human genome. Known and predicted regulatory DNA elements include regions of deoxyribonuclease hypersensitivity, binding sites of TFs, and promoter regions that have been biochemically characterized to regulate transcription. Sources of these data include public datasets from GEO, the ENCODE project, and published literature. Rank scores for common SNPs (regulomedb_dbsnp153_common_snv.tsv) were obtained from https://regulomedb.org/regulome-help/. A RegulomeDB rank of ≤2 was used to predict SNPs with the minimal functional evidence. This resulted in the identification of 524,563 functional SNPs out of the 13,275,023 referenced SNPs. Enrichments of functional SNPs at gene features were computed in 100-bp windows and normalized using background values from domains adjacent to TSSs and splice sites. Gene expression in adult male germ cells was assessed from single-cell RNA-seq analysis of whole and unfractionated testes samples from two fertile adults (*38*). Cells from the spermatogonia, spermatocyte, and spermatid lineages were used to compute gene expression levels in TPM. Mean levels of gene expression were computed by averaging TPM values obtained for different cell types and individuals. Nonexpressed genes are genes with <0.01 mean TPM values. Low-, medium-, and high-gene expression levels were computed by dividing the distribution of remaining mean expression values into three quantiles.

### TFBS enrichment

To assess the ability of constitutive origin-associated SNPs to disrupt binding of TFs, we extracted the sequence context (SNP ± 12 nt) of all functional variants, as previously defined, found within 5 kb of TSSs and identified overlapping TFBSs using the TFBSTools (*66*) R/Bioconductor packages. We used the JASPAR 2020 database (*67*) to curate a list of 639 nonredundant human TFs with associated position weight matrices (PWMs). We then analyzed each functional SNP sequence context for TFBS using the “searchSeq” function of TFBSTools using a minimum score of 95% and no strand information. TFBSs were considered as overlapping with a functional SNP if the match scores exceeded an arbitrary value of 10, and the consensus sequences defined by the PWMs contain a functional SNP. Enrichment of TFBSs at TSSs marked by origin was then tested by comparing the TFBS/SNP pair lists associated with TSS marked or free of origins. *P* values associated with the enrichment or depletion of TFBSs were computed using 2 × 2 contingency χ^2^ tests and compared to the normal expected uniform distribution to identify TFBS disrupted by constitutive origin-associated functional variants. Replication-dependent mutational loads at TFBS were then assessed by computing the number of functional variants overlapping with TFBS hits at TSSs marked by low-, medium-, and high-efficient constitutive origins.

### QTL analyses

To characterize the impact of mutagenesis at constitutive origins on human phenotypic diversity, we analyzed mutational loads for molecular cis-QTL at TSSs and splice sites marked by constitutive origins. eQTL (GTEx_Analaysis_v8_eQTL.tar) and sQTL (GTEx_Analaysis_v8_eQTL.tar) for 49 tissues were recovered from the GTEx project website at www.gtexportal.org/home/datasets. QTL/gene pairs were defined by identifying significant variant-gene associations with *q* ≥ 0.05 and cis-QTL as variants found within 5-kb domains centered at TSSs or splice sites. Cis-QTL loads were computed as the ratio of the number of significant cis-QTLs over the number of bases covered by the considered domains, i.e., TSSs and splice sites marked or free of constitutive origins. *P* values associated with the enrichment of cis-QTLs at TSSs and splice junctions marked by constitutive origins were computed using 2 × 2 contingency χ^2^ tests.

### In silico model of evolution

To determine the effect of mutagenesis at constitutive origins on their sequences, we devised an in silico model of evolution in which a library of synthetic DNA sequences was evolved according to rules defined by observed mutation processes operating at constitutive origins. We defined three PDFs that describe (i) the probability of a base to mutate according to its distance from origin centers (PDF-1; fig. S7A), (ii) the probability of the resulting mutation according to its trinucleotide context and the nature of the known mutational signatures operating at its position (PDF-2; fig. S7B), and (iii) the probability for a mutation to be fixed on the Watson or Crick strand (PDF-3; fig. S7C). The library of 20-kb DNA sequences contained 1000 sequences calibrated on noncoding upstream sequences from the hg38 version of the human genome and using a Markov model with an oligonucleotide size of 6 (rsat.sb-roscoff.fr/random-seq_form.cgi). PDF-1 was constructed by fitting a multipeak Gaussian profile to the observed mutation rates reported in Fig. 1D. PDF-2 was defined by fitting the contribution of known signatures of somatic mutations in noncancerous and cancerous human tissues (COSMIC v3.2) using an SNP mutation count matrix, describing mutagenesis at constitutive origins, corrected by mononucleotide and trinucleotide composition. This corrects for the excess of base substitutions that are due to compositional biases and allows modeling the impact of replication initiation on “naïve” sequences. Mutational signature exposures were then fitted using a combination of Gaussian or sigmoidal functions according to the observed profiles. PDF-3 was constructed by fitting a combination of Gaussian or sigmoidal profiles to the observed replication strand biases reported in Fig. 1G. We then apply 5000 rounds of evolution to the DNA library by using PDF-1 to select 100 bases to mutate and PDF-2 and PDF-3 to output the set of mutated sequences. The in silico evolution process was stopped when values associated with general features of constitutive origins, such as the GC content, plateaued.

### Sequence features

DNA sequence analysis was performed using the seqinr (*68*) and Biostrings (*69*) R/Bioconductor packages. Nucleotide composition, nucleotide skews, and diversity indexes were computed in 100-bp windows spanning 20 kb centered on origin midpoints. AT and GC skews were computed as follows: ${\text{AT}}_{\text{skew}}=\frac{A-T}{A+T}\;\text{and}\;{\text{GC}}_{\text{skew}}=\frac{G-C}{G+C}$, where A, T, C, and G are the number of the corresponding nucleotides in the considered window. G-quadruplexes were predicted by considering G_3+_N_1–7_G_3+_N_1–7_G_3+_N_1–7_G_3+_, where N refers to any bases, regular expression (*70*). Shannon (or Shannon-Wiener) diversity indices (SDIs) were computed by considering the occurrence of each of the *k*-mers (*k* = 6) and the following equation: $\text{SDI}=\sum_{i=1}^{4096}p_{i}.\text{log}(p_{i}),$ where *p_i_* is the proportional abundance of one of the 4096 6-mers. As SDI values depend on the number of sequences analyzed, computed values were corrected to account for different sample size when appropriate.

### Human conservation measures

GERP scores (*44*) were used as a measure of nucleotide diversity between species. The single-nucleotide resolution bigWig file (gerp_conservation_scores.homo_sapiens.GRCh38.bw), reporting substitution rates computed from the multiple alignment of 111 mammalian genomes, was obtained from ftp://ftp.ensembl.org/pub/release101/compara/conservation_scores/111_mammals.gerp_conservation_score/. GERP scores were computed as averages from rolling windows of 100 nt spanning 20 kb centered on origin midpoints. For consistency of presentation with plots of mutation rates and sequence diversity, the *y* axes in plots showing GERP scores have been inverted so that greater constraint is low and greater diversity is high.

### Computational and statistical analyses

Analysis and all statistical calculations were performed in R (version 4.0.3).

## Supplementary Materials

### This PDF file includes:

Figs. S1 to S7 Table S1 References

## Notes

View/request a protocol for this paper from *Bio-protocol*.
