# Supplementary material for: DNA replication initiation shapes the mutational landscape and expression of the human genome
Source: Sci Adv. 2022 Nov 9;8(45):eadd3686. doi: 10.1126/sciadv.add3686 (PMC9645720; doi:10.1126/sciadv.add3686)
Supplement: Supplementary file 1 — Figs. S1 to S7 Table S1 References [file sciadv.add3686_sm.pdf]

Science Advances
AAAS

Supplementary Materials for

DNA replication initiation shapes the mutational landscape and expression of the human genome

Pierre Murat *et al.*

Corresponding author: Pierre Murat, pmurat@mrc-lmb.cam.ac.uk; Julian E. Sale, jes@mrc-lmb.cam.ac.uk

Sci. Adv. **8**, eadd3686 (2022)
DOI: 10.1126/sciadv.add3686

This PDF file includes:

Figs. S1 to S7
Table S1
References

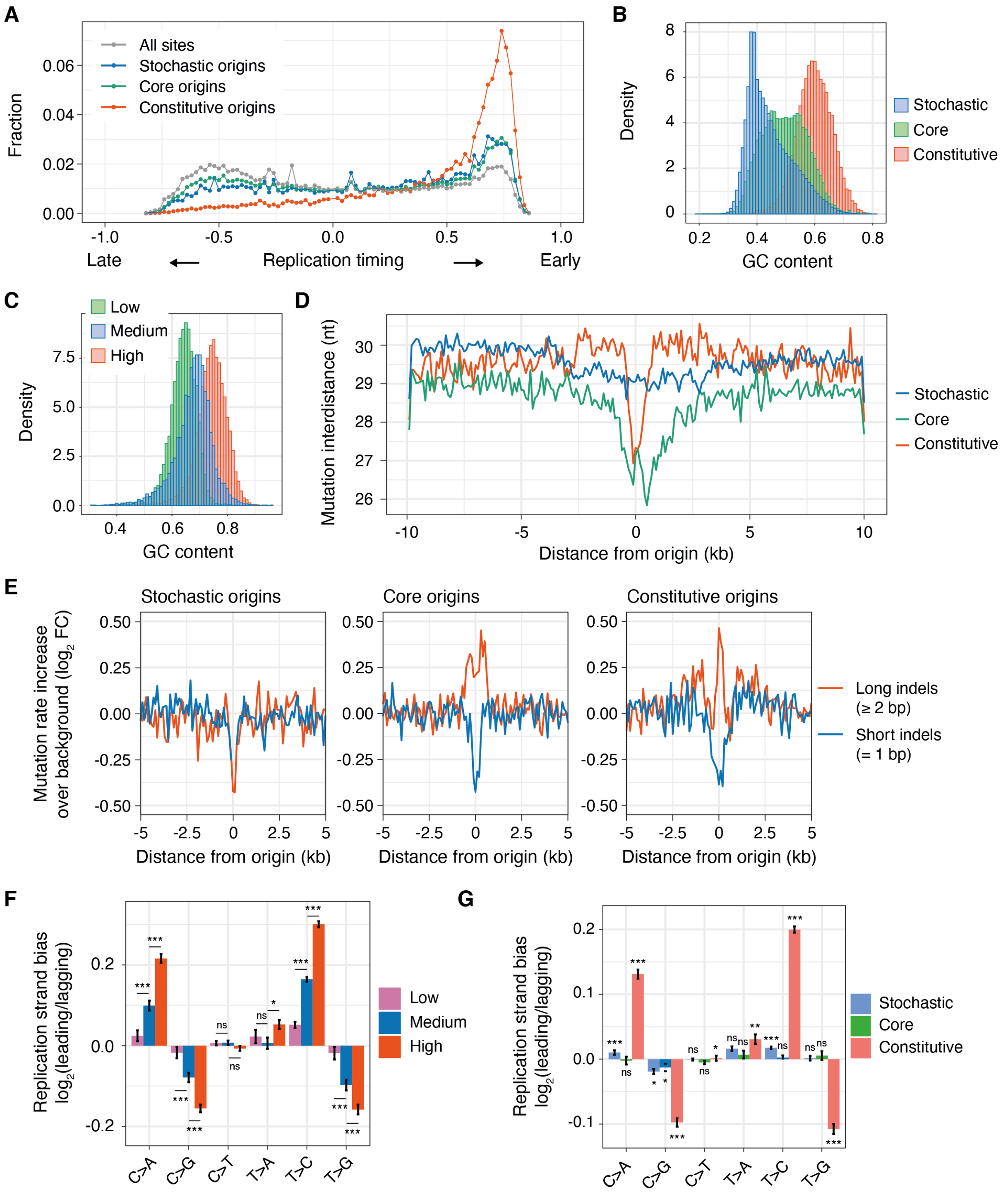

Fig. S1. Constitutive origins are hotspots for replication-dependent mutagenesis. (A) Human replication origins were categorised as stochastic and core origins according to their human cell types specificity (see Methods). A subset of core origins, mapped by initiation site sequencing (ini-seq 2), were found to be enriched within early replicating domains of the human genome. Origin base composition determines origin usage **(B)** and efficiency **(C)**. Origins considered in panel **(C)** are subsets of constitutive origins from panel **(B)** binned in three groups of equal sizes and different efficiencies. The density plots, reporting the GC content of 1 kb or 100 bp, for panel **(B)** and **(C)** respectively, sequences centred on origins, show that constitutive origins display higher GC contents than core and constitutive origins and that highly-efficient origins are more GC rich that the less efficient ones. **(D)** Local

decrease in SNP interdistances at origins compared with their flanking domains ($P = 1.68 \times 10^{-8}$, 7.79×10^{-24} and 1.15×10^{-5} for constitutive, core and stochastic origins respectively, chi-square test). Mutation interdistances were computed for each mutation as the smallest distance between the first upstream or downstream mutation and we averaged values for all variants in 100 bp windows covering origin domains. **(E)** Mutation rates for long (≥ 2 bp) but not short (= 1bp) indels is increased at core and constitutive origins compared to their flanking domains ($P = 2.82 \times 10^{-5}$ and 3.73×10^{-4} for constitutive and core origins respectively, chi-square test). Mutation rates are reported as the increase over background values computed from domains adjacent to origins. Replication strand biases for variants at origins are efficiency- **(F)** and usage-**(G)** dependent. Replication strand biases were computed, as the ratio between the density of mutation at a given base pair over the density of mutation at the complementary base pair in 100 bp windows covering 20 kb origin domains as in Fig. 1g. We then computed the ratio between averaged values for 10 kb domains covering leading- (left of origins) over lagging- (right of origins) strand synthesis. *P* values for the comparison of the distribution of individual values computed in 100 bp windows were calculated using the Kolmogorov-Smirnov test, *n.s.* non-significant, $^*P < 0.05$, $^{**}P < 0.01$ and $^{***}P < 0.001$.

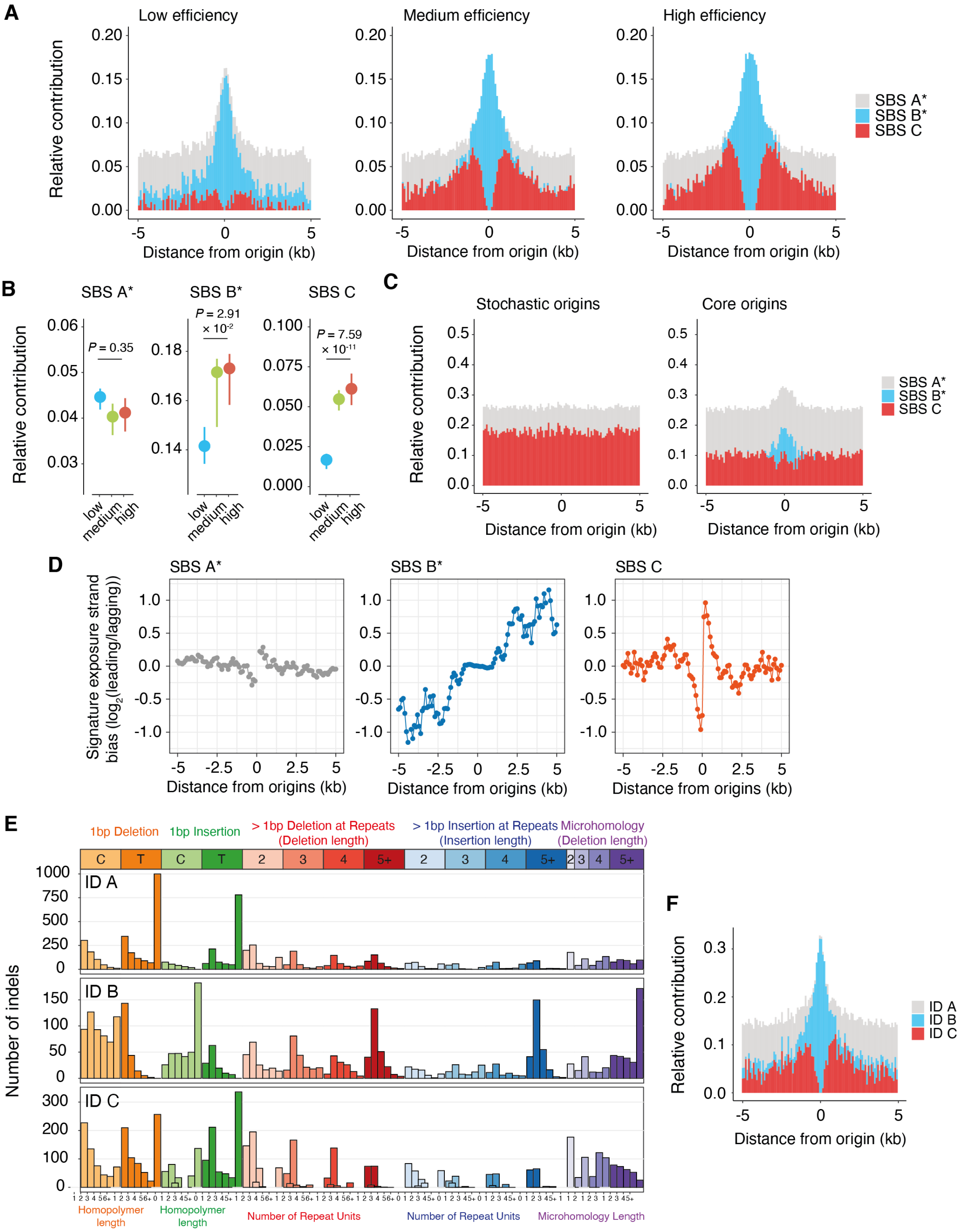

Fig. S2. Replication-dependent mutational processes operating at constitutive origins. (A) The contribution of the *de novo* extracted SBS B* and C signatures to mutagenesis at constitutive origins correlates with origin efficiency. Reporting the relative contribution of SBS A*, B* and C at constitutive origins binned as low-, medium- and high-efficient origins shows that while the contribution of SBS A* remain constant over origin domains, the absolute contribution of SBS B* and C increases with origin efficiency. **(B)** To assess the replication-

dependency of SBS A*, B* and C, we considered the relative contribution of each signature to domains around origins where the signatures operate. We considered 1 kb domains, 4 kb domains but omitting the previous domains and 16 kb domains excluding the previous ones centred at the origins for SBS B*, C and A* respectively. Reported *P* values are from chi-square tests of independence assessing the impact of origin efficiency on SBS A*, B* and C contribution. **(C)** The contribution of SBS B* and C to mutagenesis at origins reflects origin usage with any contribution to mutagenesis at stochastic and core origins largely swamped by background signal from SBS A*. **(D)** Replication strand bias for exposures associated with signatures SBS A*, B* and C. **(E)** An unbiased *de novo* mutational signature analysis identified three small insertion/deletion (indel) signatures, ID A-C, operating at constitutive origins. Each profile reports the number of mutations attributed to each indicated indels type. ID A and C signatures are characterised by insertions and deletions at long (≥ 5 bp) homopolymers. ID B signature is characterised by increased long (≥ 5 bp) deletions with at least 5 bp of microhomology at their boundaries. **(F)** Computing the relative contribution of ID A-C to mutagenesis at constitutive origins shows that ID A-C operate in concert with SBS A-C as they present similar profile (see **Fig. 2D**).

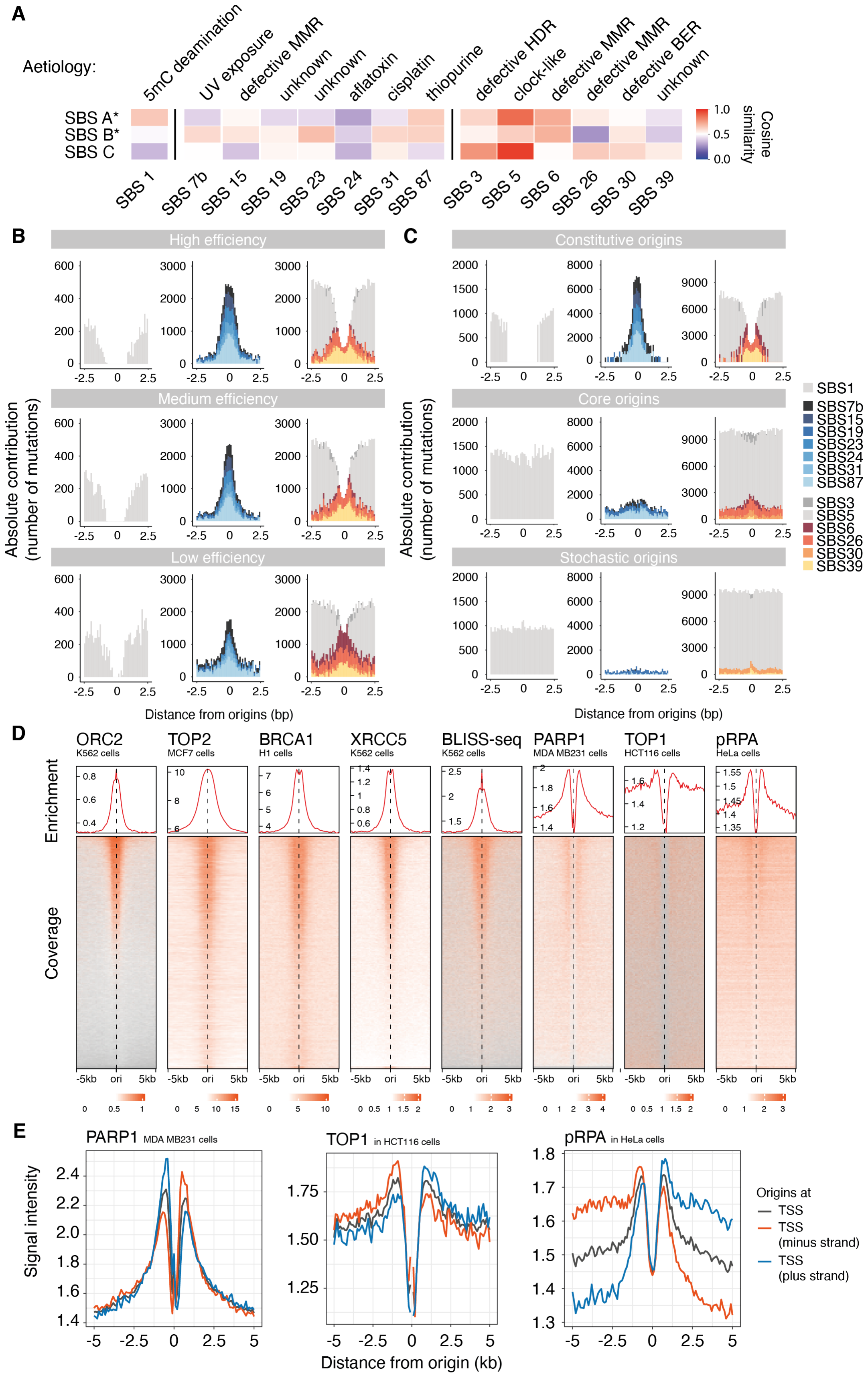

Fig. S3. Characterisation of the molecular mechanisms underlying mutagenesis at constitutive origins. Known signatures of somatic mutations in noncancerous and cancerous human tissues, collected by the Catalogue of Somatic Mutations in Cancer (COSMIC v3.2),

were fitted to mutational count matrixes computed from the frequencies of SNPs observed at constitutive origins. We used an iterative fitting approach to select the minimum set of mutational processes operating at constitutive origins and avoid signature misattribution. We identified a set of 14 known signatures that mostly reconstruct the mutation profiles of SBS A*, B* and C. **(A)** Comparison of the *de novo* extracted SBS A*, B* and C signatures to the set of 14 known signatures based on cosine similarity. These signatures were assigned to one of the SBS A*, B* or C signature according to their similarity and exposure. Exposure of origins to the mutational processes defined by these SBS signatures is replication-dependent as their contribution to origin mutagenesis is efficiency- **(B)** and usage- **(C)** dependent. We plotted the absolute contribution of the 14 known signatures split in three groups to highlight the three mutational processes described by SBS A*, B* and C (left, middle and right columns show the contribution of SBS signatures reconstructing the profiles of SBS A*, B* and C respectively). **(D)** Concatenated heatmaps for DNA repair factors binding at constitutive origins. The rows of each heatmap are ordered by decreasing ORC2 signal. Such representation allows analysing all signals in parallel at the same sites. We observe that DNA repair factors and DSBs bind and occur concomitantly at origins and do not represent independent events. **(E)** Enrichment of PARP1, TOP1 and pRPA at constitutive origins found within ± 5kb of TSSs and oriented by promoter direction. This analysis shows that the 'volcano-shaped' enrichment pattern associated with these factors can not be explained by the direction of transcription.

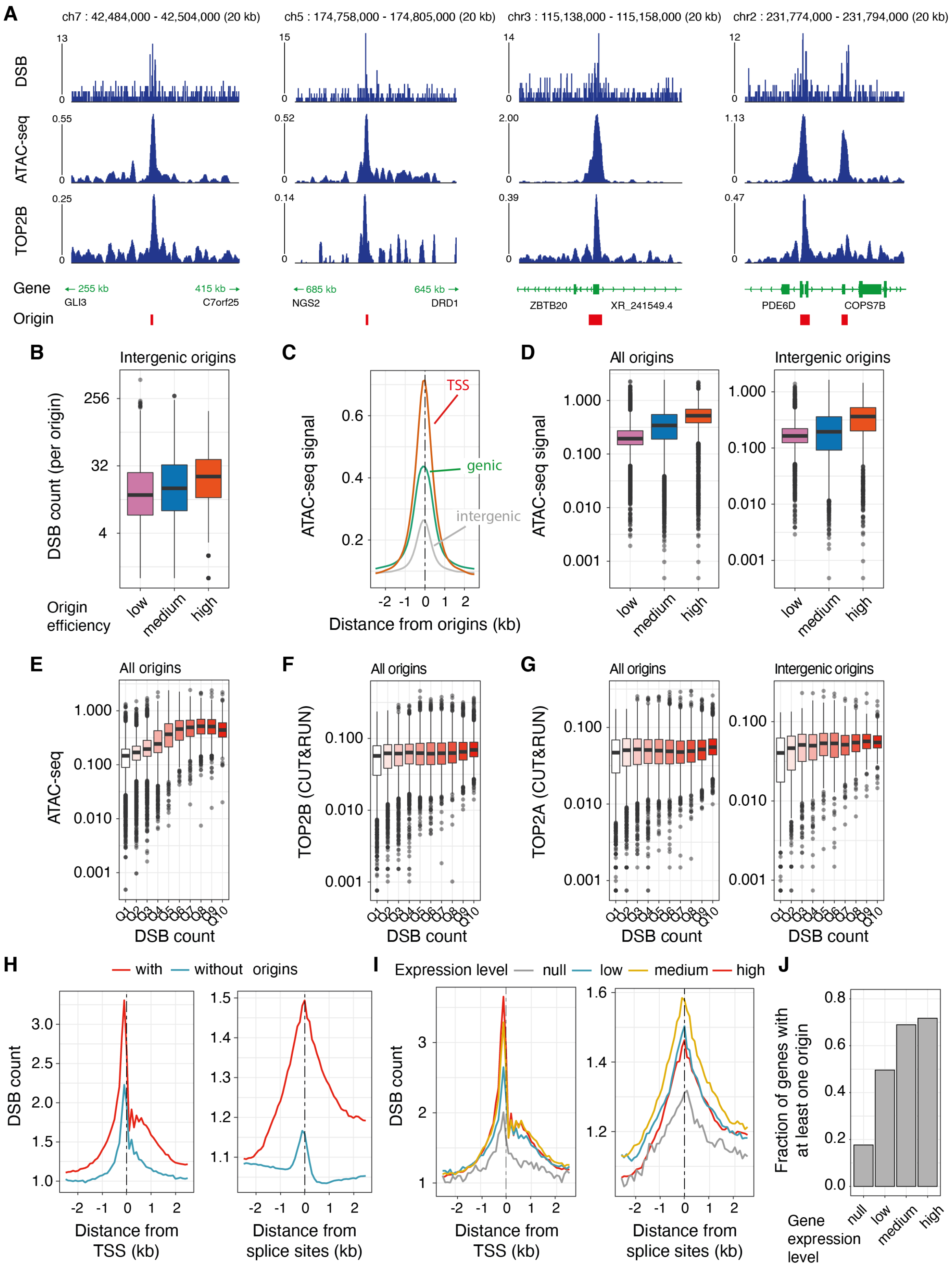

Fig. S4. Genomic characterisation of constitutive origins in human H9 embryonic stem cells. **(A)** Representative sequencing tracks showing the accumulation of DSBs, increased DNA accessibility and enrichment of TOP2B at constitutive origins (red boxes). Sequencing data is from Induce-seq, ATAC-seq and TOP2B CUT&RUN assays. Examples of intergenic (left panels) and genic (right panels) origins were selected to illustrate that DSBs accumulate

at constitutive origins independently of their environment. **(B)** DSB counts at intergenic origins correlates with origin efficiency ($P < 2.2 \times 10^{-16}$). **(C)** Constitutive origin sequences are characterised by an increase in accessibility as seen by a local increase in ATAC-seq signal intensity. **(D)** ATAC-seq signal intensity at all (left panel) of intergenic (right panel) origins correlate with origin efficiency ($P < 2.2 \times 10^{-16}$). DSB counts at constitutive origins correlates with **(E)** ATAC-seq ($P < 2.2 \times 10^{-16}$), **(F)** TOP2B ($P = 2.64 \times 10^{-2}$) and **(G)** TOP2A ($P = 1.19 \times 10^{-2}$ and 7.46×10^{-3} for all and intergenic origins respectively) signal intensities. DSB counts are binned by Q1-10 quantiles with higher counts in Q10. *P* values were calculated using chi-square tests of independence. **(H)** The presence of constitutive origins increases the number of DSBs at gene TSSs (left panel) and splice sites (right panel). Protein coding genes were categorized according to the presence (n = 11,410) or absence (n = 7,925) of constitutive origins within 2.5 kb around their TSS. Splice sites of GENCODE basic transcripts were categorized according to the presence (n = 55,903) or absence (n = 121,511) of constitutive origins within 2.5 kb. DSB counts were computed in bins of 100 bp and normalised using background values from adjacent domains. **(I)** DSB densities at these gene features also depend on gene expression. TSSs and splice sites were categorized by gene expression level using RNA-seq data from H9 cells. (**J**) Fraction of genes that host at least one constitutive origin within its body. Gene expression levels were computed as in **Fig. 4I**.

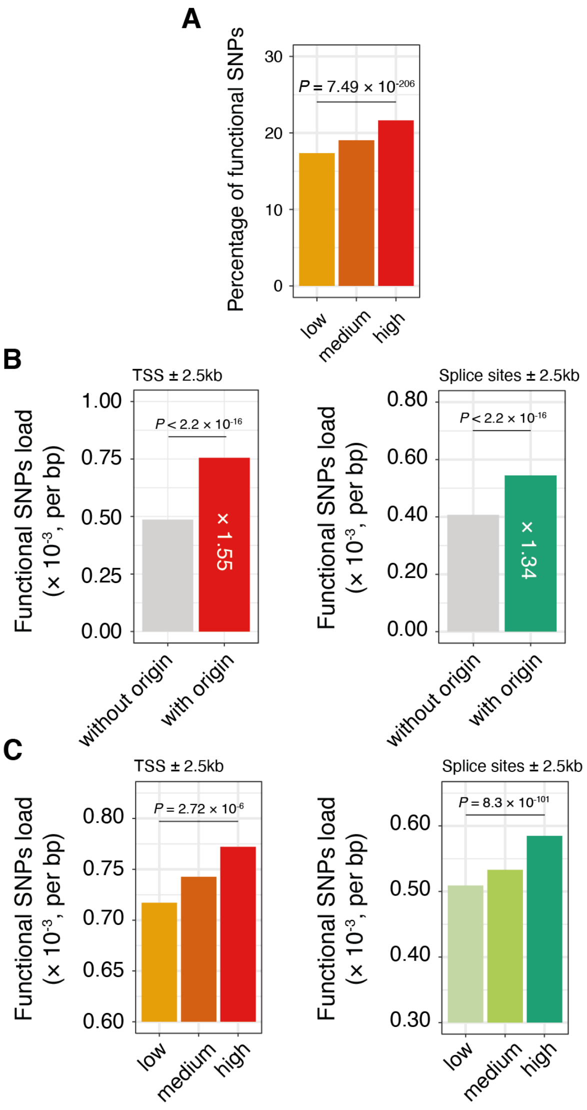

Fig. S5. Constitutive origins increase functional variants load at TSSs and splice sites. (A) The percentage of functional SNPs associated with constitutive origins is replication-dependent as it correlates with origin efficiency. **(B)** The presence of constitutive origins increases mutational loads associated with functional variants at gene TSSs and splice sites. Quantification of functional SNPs load was performed considering 5 kb domains centred on either TSSs or splice sites. **(C)** The percentage of functional SNPs associated with constitutive origins at TSSs and splice sites is replication-dependent as it correlates with origin efficiency. *P* values were calculated using chi-square tests of independence.

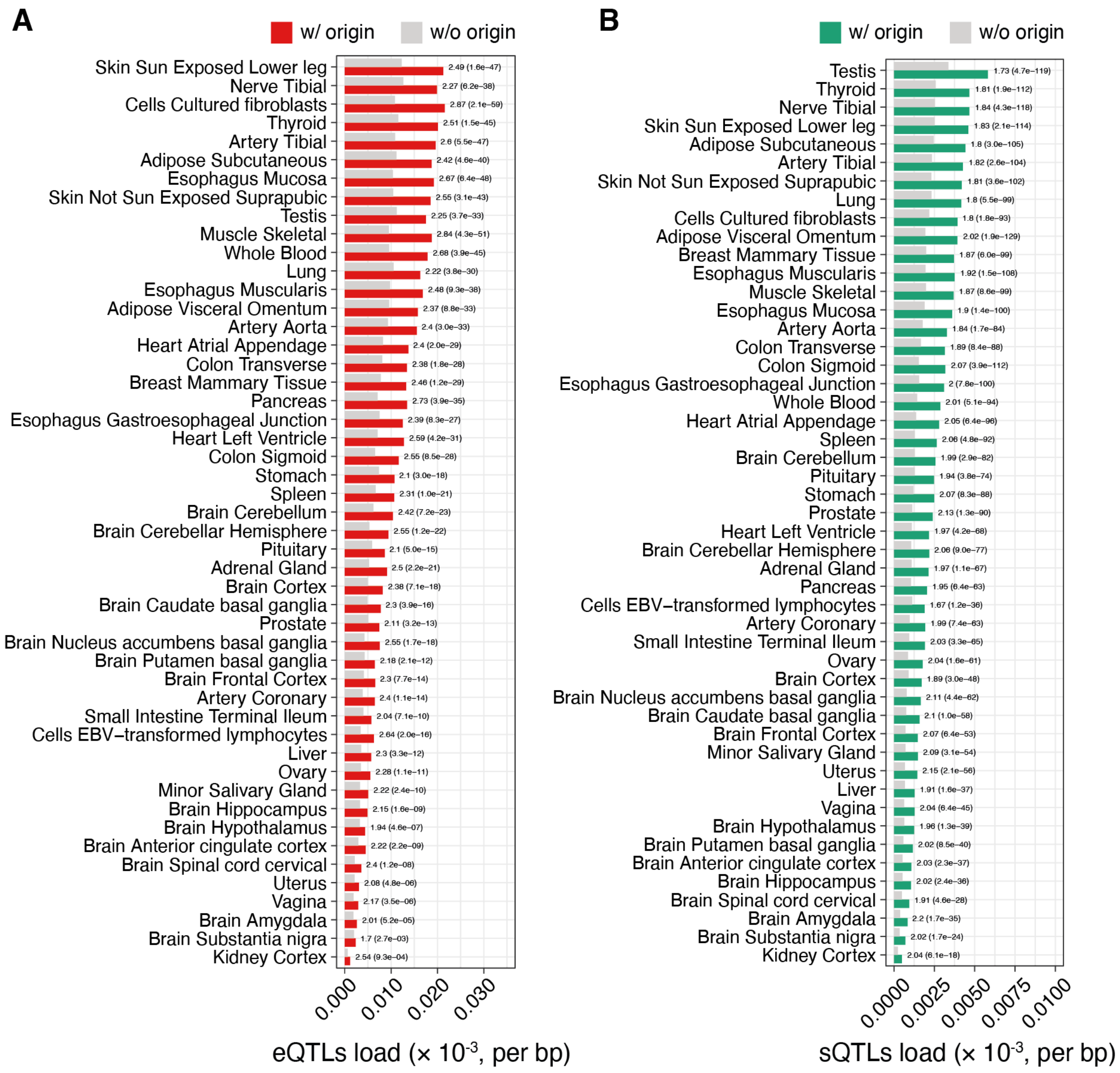

Fig. S6. Mutagenesis at constitutive origins drive gene expression variation and alternative splicing in humans. (A) *cis*-expression quantitative trait loci (*cis*-eQTL) and **(B)** *cis*-splicing quantitative trait loci (*cis*-sQTL) densities within 5 kb regions centred on gene TSSs or splice sites when computed for features marked by constitutive origins (red and green bars for TSSs and splice sites respectively) or free from origins (grey bars) in 49 human tissues together with enrichment values. This panel reports the full analysis related to **Fig. 6**. *P* values were computed using chi-square tests.

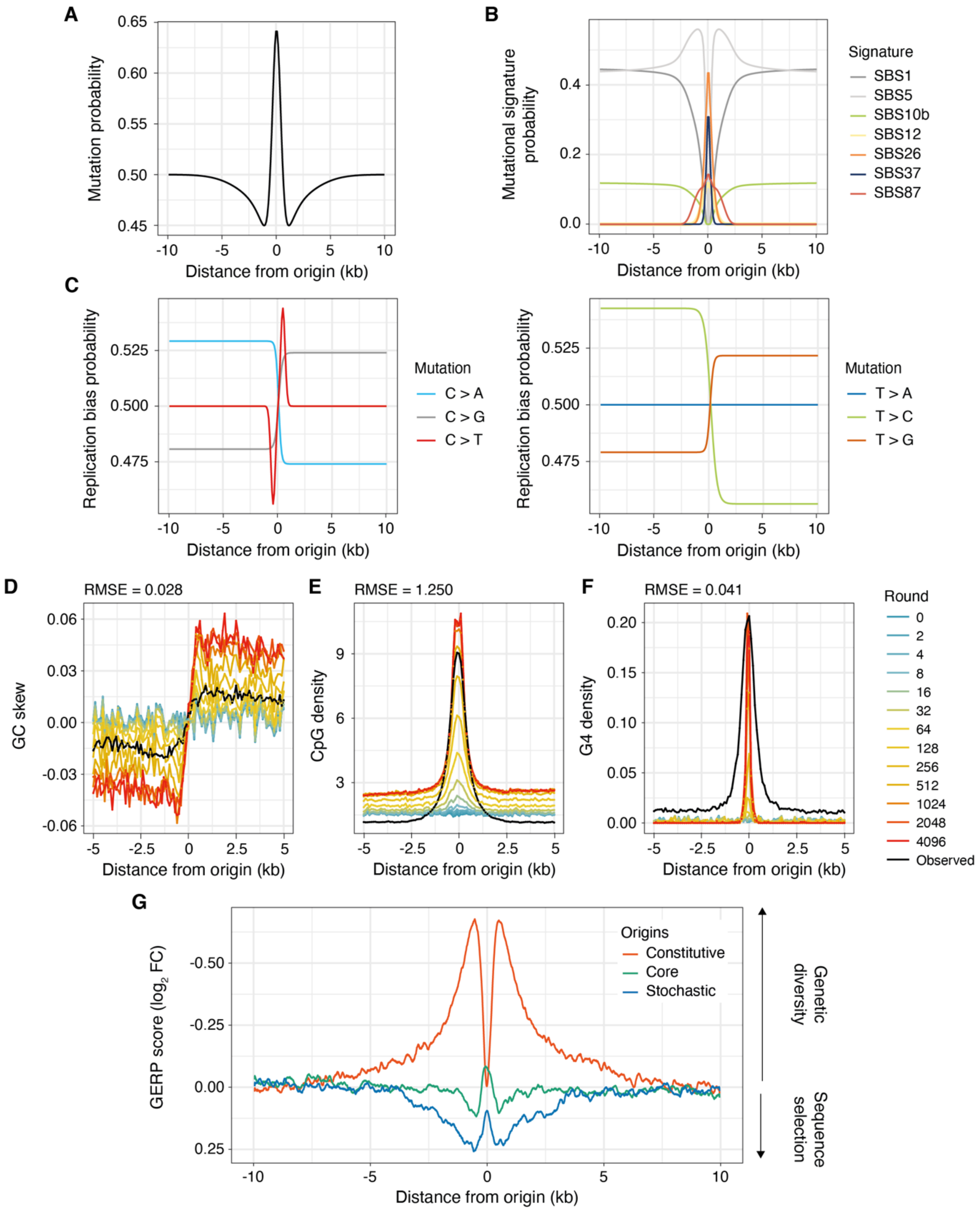

Fig. S7. An in silico model of evolution recapitulates general features of constitutive origins. To determine the effect of mutagenesis at constitutive origins on origin sequences, we devised an in silico model of evolution in which a library of synthetic DNA sequences was evolved according to rules defined by observed mutation processes operating at constitutive origins. We defined three probability density functions (PDFs) that describe **(A)** the probability of a base to mutate according to its distance from the origin centre, **(B)** the probability of the resulting mutation according to its trinucleotide context and the nature of the known mutational signatures operating at its position and **(C)** the probability for a mutation to be fixed on the top or bottom strand (See Methods section for details). A library of 1,000 × 20 kb DNA sequences

calibrated on non-coding upstream sequences from the human genome were evolved using these PDFs to select the bases to mutate and output the resulting mutated sequences. 5,000 rounds of in-silico evolution allow recapitulation of the features of constitutive origins such as **(D)** GC-skews, **(E)** CpG and **(F)** G-quadruplexes (G4s) density. Coloured lines represent values at different rounds of evolution and black lines correspond to values observed at constitutive origins. The Root Mean Square Error (RMSE) values are the standard deviations of the residuals between computed values at round 4,096 and observed values. g, Rates of genome evolution, plotted as GERP scores, at constitutive, core and stochastic origins. GERP scores were normalised using background values from domains adjacent to origins. Positive and negative GERP scores indicate an increase and decrease in nucleotide substitution rates relative to a genome-wide expectation of neutral evolution respectively.

Table S1. Gene Expression Omnibus (GEO) accession numbers and associated references for the datasets used in the analyses reported in **Fig. 3B-D**.

**Target**	**Cell line**	**GEO accession numbers**	**GSM accession numbers**	**References**
ORC1	HeLa S3	GSE37583	GSM922790	(*71*)
ORC2	K562	GSE70165	GSM1717888	(*72*)
TOP2B	MCF7	GSE141528	GSM4205700	(*73*)
TOP2B	MCF10	GSE93038	GSM2442946	(*74*)
BLISS	TK6	GSE121740	GSM3444984	(*75*)
BLISS	K562	GSE121740	GSM3444988	(*75*)
BLISS	CD34	GSE121740	GSM3687236	(*75*)
BRCA1	MCF10A	GSE40591	GSM1340576	(*76*)
BRCA1	H1	GSE31477	GSM935517	ENCODE Project
BRCA1	HeLa	GSE51334	GSM935552	ENCODE Project
BRCA1	GM12878	GSE31477	GSM935377	ENCODE Project
XRCC5	K562	GSE120110	GSM3393608	ENCODE Project
XRCC5	HepG2	GSM3391789	GSM3393560	(*77*)
RAD51	MCF10A	GSE93038	GSM2442936	(*74*)
TOP1	MCF10A	GSE93038	GSM3182665	(*74*)
TOP1	HCT116	GSE57628	GSM2058666	(*78*)
TOP-seq	HCT116	GSE57628	GSM1385717	(*78*)
PARP1	MCF10	GSE93038	GSM3182666	(*74*)
PARP1	MCF7	GSE61916	GSM1517305	(*79*)
PARP1	MDA-MB231	GSE61916	GSM1517306	(*79*)
RPA1	HeLa	GSE76661	GSM2033106	(*80*)
RPA2	HeLa	GSE76661	GSM2033104	(*80*)
phospho-RPA2-S33	HeLa	GSE108172	GSM2891676	(*81*)

REFERENCES AND NOTES

1. J. A. Stamatoyannopoulos, I. Adzhubei, R. E. Thurman, G. V. Kryukov, S. M. Mirkin, S. R. Sunyaev, Human mutation rate associated with DNA replication timing. *Nat. Genet.* **41**, 393–395 (2009).

2. B. Schuster-Böckler, B. Lehner, Chromatin organization is a major influence on regional mutation rates in human cancer cells. *Nature* **488**, 504–507 (2012).

3. M. Y. Tolstorukov, N. Volfovsky, R. M. Stephens, P. J. Park, Impact of chromatin structure on sequence variability in the human genome. *Nat. Struct. Mol. Biol.* **18**, 510–515 (2011).

4. O. Pich, F. Muiños, R. Sabarinathan, I. Reyes-Salazar, A. Gonzalez-Perez, N. Lopez-Bigas, Somatic and germline mutation periodicity follow the orientation of the DNA minor groove around nucleosomes. *Cell* **175**, 1074–1087.e18 (2018).

5. M. A. M. Reijns, H. Kemp, J. Ding, S. M. de Procé, A. P. Jackson, M. S. Taylor, Lagging-strand replication shapes the mutational landscape of the genome. *Nature* **518**, 502–506 (2015).

6. R. Sabarinathan, L. Mularoni, J. Deu-Pons, A. Gonzalez-Perez, N. López-Bigas, Nucleotide excision repair is impaired by binding of transcription factors to DNA. *Nature* **532**, 264–267 (2016).

7. D. Perera, R. C. Poulos, A. Shah, D. Beck, J. E. Pimanda, J. W. Wong, Differential DNA repair underlies mutation hotspots at active promoters in cancer genomes. *Nature* **532**, 259–263 (2016).

8. A. Gonzalez-Perez, R. Sabarinathan, N. Lopez-Bigas, Local determinants of the mutational landscape of the human genome. *Cell* **177**, 101–114 (2019).

9. M. E. Douglas, F. A. Ali, A. Costa, J. F. X. Diffley, The mechanism of eukaryotic CMG helicase activation. *Nature* **555**, 265–268 (2018).

10. T. A. Guilliam, J. T. P. Yeeles, An updated perspective on the polymerase division of labor during eukaryotic DNA replication. *Crit. Rev. Biochem. Mol. Biol.* **55**, 1–13 (2020).

11. I. Akerman, B. Kasaai, A. Bazarova, P. B. Sang, I. Peiffer, M. Artufel, R. Derelle, G. Smith, M. Rodriguez-Martinez, M. Romano, S. Kinet, P. Tino, C. Theillet, N. Taylor, B. Ballester, M. Méchali, A predictable conserved DNA base composition signature defines human core DNA replication origins. *Nat. Commun.* **11**, 4826 (2020).

12. G. Guilbaud, P. Murat, H. S. Wilkes, L. K. Lerner, J. E. Sale, T. Krude, Determination of human DNA replication origin position and efficiency reveals principles of initiation zone organisation. *Nucleic Acids Res.* **50**, 7436–7450 (2022).

13. H. Jónsson, P. Sulem, B. Kehr, S. Kristmundsdottir, F. Zink, E. Hjartarson, M. T. Hardarson, K. E. Hjorleifsson, H. P. Eggertsson, S. A. Gudjonsson, L. D. Ward, G. A. Arnadottir, E. A. Helgason, H. Helgason, A. Gylfason, A. Jonasdottir, A. Jonasdottir, T. Rafnar, M. Frigge, S. N. Stacey, O. Th Magnusson, U. Thorsteinsdottir, G. Masson, A. Kong, B. V. Halldorsson, A. Helgason, D. F. Gudbjartsson, K. Stefansson, Parental influence on human germline de novo mutations in 1,548 trios from Iceland. *Nature* **549**, 519–522 (2017).

14. F. Blokzijl, R. Janssen, R. van Boxtel, E. Cuppen, MutationalPatterns: Comprehensive genome-wide analysis of mutational processes. *Genome Med.* **10**, 33 (2018).

15. E. A. Sia, S. Jinks-Robertson, T. D. Petes, Genetic control of microsatellite stability. *Mutat. Res.* **383**, 61–70 (1997).

16. H. H. Y. Chang, N. R. Pannunzio, N. Adachi, M. R. Lieber, Non-homologous DNA end joining and alternative pathways to double-strand break repair. *Nat. Rev. Mol. Cell Biol.* **18**, 495–506 (2017).

17. P. J. Hastings, G. Ira, J. R. Lupski, A microhomology-mediated break-induced replication model for the origin of human copy number variation. *PLOS Genet.* **5**, e1000327 (2009).

18. L. B. Alexandrov, J. Kim, N. J. Haradhvala, M. N. Huang, A. W. Tian Ng, Y. Wu, A. Boot, K. R. Covington, D. A. Gordenin, E. N. Bergstrom, S. M. A. Islam, N. Lopez-Bigas, L. J.

Klimczak, J. R. McPherson, S. Morganella, R. Sabarinathan, D. A. Wheeler, V. Mustonen; PCAWG Mutational Signatures Working Group, G. Getz, S. G. Rozen, M. R. Stratton; PCAWG Consortium, The repertoire of mutational signatures in human cancer. *Nature* **578**, 94–101 (2020).

19. L. B. Alexandrov, P. H. Jones, D. C. Wedge, J. E. Sale, P. J. Campbell, S. Nik-Zainal, M. R. Stratton, Clock-like mutational processes in human somatic cells. *Nat. Genet.* **47**, 1402–1407 (2015).

20. D. Cortez, Replication-coupled DNA repair. *Mol. Cell* **74**, 866–876 (2019).

21. D. Remus, E. L. Beall, M. R. Botchan, DNA topology, not DNA sequence, is a critical determinant for Drosophila ORC-DNA binding. *EMBO J.* **23**, 897–907 (2004).

22. G. Abdurashidova, S. Radulescu, O. Sandoval, S. Zahariev, M. B. Danailov, A. Demidovich, L. Santamaria, G. Biamonti, S. Riva, A. Falaschi, Functional interactions of DNA topoisomerases with a human replication origin. *EMBO J.* **26**, 998–1009 (2007).

23. H. G. Hu, M. Baack, R. Knippers, Proteins of the origin recognition complex (ORC) and DNA topoisomerases on mammalian chromatin. *BMC Mol. Biol.* **10**, 36 (2009).

24. E. Rampakakis, M. Zannis-Hadjopoulos, Transient dsDNA breaks during pre-replication complex assembly. *Nucleic Acids Res.* **37**, 5714–5724 (2009).

25. W. X. Yan, R. Mirzazadeh, S. Garnerone, D. Scott, M. W. Schneider, T. Kallas, J. Custodio, E. Wernersson, Y. Li, L. Gao, Y. Federova, B. Zetsche, F. Zhang, M. Bienko, N. Crosetto, BLISS is a versatile and quantitative method for genome-wide profiling of DNA double-strand breaks. *Nat. Commun.* **8**, 15058 (2017).

26. S. P. Jackson, Sensing and repairing DNA double-strand breaks. *Carcinogenesis* **23**, 687–696 (2002).

27. G. Jiang, I. Plo, T. Wang, M. Rahman, J. H. Cho, E. Yang, B. S. Lopez, F. Xia, BRCA1-Ku80 protein interaction enhances end-joining fidelity of chromosomal double-strand breaks in the G1 phase of the cell cycle. *J. Biol. Chem.* **288**, 8966–8976 (2013).

28. J. Schimmel, H. Kool, R. van Schendel, M. Tijsterman, Mutational signatures of non-homologous and polymerase theta-mediated end-joining in embryonic stem cells. *EMBO J.* **36**, 3634–3649 (2017).

29. M. Shyian, B. Albert, A. M. Zupan, V. Ivanitsa, G. Charbonnet, D. Dilg, D. Shore, Fork pausing complex engages topoisomerases at the replisome. *Genes Dev.* **34**, 87–98 (2020).

30. M. A. Garbacz, S. A. Lujan, A. B. Burkholder, P. B. Cox, Q. Wu, Z. X. Zhou, J. E. Haber, T. A. Kunkel, Evidence that DNA polymerase δ contributes to initiating leading strand DNA replication in Saccharomyces cerevisiae. *Nat. Commun.* **9**, 858 (2018).

31. V. Aria, J. T. P. Yeeles, Mechanism of bidirectional leading-strand synthesis establishment at eukaryotic DNA replication origins. *Mol. Cell* **73**, 199–211.e10 (2018).

32. Z. X. Zhou, S. A. Lujan, A. B. Burkholder, M. A. Garbacz, T. A. Kunkel, Roles for DNA polymerase δ in initiating and terminating leading strand DNA replication. *Nat. Commun.* **10**, 3992 (2019).

33. P. S. Robinson, T. H. H. Coorens, C. Palles, E. Mitchell, F. Abascal, S. Olafsson, B. C. H. Lee, A. R. J. Lawson, H. Lee-Six, L. Moore, M. A. Sanders, J. Hewinson, L. Martin, C. M. A. Pinna, S. Galavotti, R. Rahbari, P. J. Campbell, I. Martincorena, I. Tomlinson, M. R. Stratton, Increased somatic mutation burdens in normal human cells due to defective DNA polymerases. *Nat. Genet.* **53**, 1434–1442 (2021).

34. E. Olson, C. J. Nievera, V. Klimovich, E. Fanning, X. Wu, RPA2 is a direct downstream target for ATR to regulate the S-phase checkpoint. *J. Biol. Chem.* **281**, 39517–39533 (2006).

35. V. M. Vassin, R. W. Anantha, E. Sokolova, S. Kanner, J. A. Borowiec, Human RPA phosphorylation by ATR stimulates DNA synthesis and prevents ssDNA accumulation during DNA-replication stress. *J. Cell Sci.* **122**, 4070–4080 (2009).

36. F. M. Dobbs, P. van Eijk, M. D. Fellows, L. Loiacono, R. Nitsch, S. H. Reed, Precision digital mapping of endogenous and induced genomic DNA breaks by INDUCE-seq. *Nat. Commun.* **13**, 3989 (2022).

37. A. P. Boyle, E. L. Hong, M. Hariharan, Y. Cheng, M. A. Schaub, M. Kasowski, K. J. Karczewski, J. Park, B. C. Hitz, S. Weng, J. M. Cherry, M. Snyder, Annotation of functional variation in personal genomes using RegulomeDB. *Genome Res.* **22**, 1790–1797 (2012).

38. A. Sohni, K. Tan, H. W. Song, D. Burow, D. G. de Rooij, L. Laurent, T. C. Hsieh, R. Rabah, S. S. Hammoud, E. Vicini, M. F. Wilkinson, The neonatal and adult human testis defined at the single-cell level. *Cell Rep.* **26**, 1501–1517.e4 (2019).

39. D. Eckert, S. Buhl, S. Weber, R. Jäger, H. Schorle, The AP-2 family of transcription factors. *Genome Biol.* **6**, 246 (2005).

40. J. Kaczynski, T. Cook, R. Urrutia, Sp1- and Krüppel-like transcription factors. *Genome Biol.* **4**, 206 (2003).

41. GTEx Consortium, Genetic effects on gene expression across human tissues. *Nature* **550**, 204–213 (2017).

42. C. Cayrou, P. Coulombe, A. Vigneron, S. Stanojcic, O. Ganier, I. Peiffer, E. Rivals, A. Puy, S. Laurent-Chabalier, R. Desprat, M. Méchali, Genome-scale analysis of metazoan replication origins reveals their organization in specific but flexible sites defined by conserved features. *Genome Res.* **21**, 1438–1449 (2011).

43. E. Besnard, A. Babled, L. Lapasset, O. Milhavet, H. Parrinello, C. Dantec, J. M. Marin, J. M. Lemaitre, Unraveling cell type-specific and reprogrammable human replication origin signatures associated with G-quadruplex consensus motifs. *Nat. Struct. Mol. Biol.* **19**, 837–844 (2012).

44. G. M. Cooper, E. A. Stone, G. Asimenos, C. S. P. NISC, E. D. Green, S. Batzoglou, A. Sidow, Distribution and intensity of constraint in mammalian genomic sequence. *Genome Res.* **15**, 901–913 (2005).

45. V. B. Seplyarskiy, R. A. Soldatov, E. Koch, R. J. McGinty, J. M. Goldmann, R. D. Hernandez, K. Barnes, A. Correa, E. G. Burchard, P. T. Ellinor, S. T. McGarvey, B. D. Mitchell, R. S. Vasan, S. Redline, E. Silverman, S. T. Weiss, D. K. Arnett, J. Blangero, E. Boerwinkle, J. He, C. Montgomery, D. C. Rao, J. I. Rotter, K. D. Taylor, J. A. Brody, Y. I. Chen, L. de Las Fuentes, C. M. Hwu, S. S. Rich, A. W. Manichaikul, J. C. Mychaleckyj, N. D. Palmer, J. A. Smith, S. L. R. Kardia, P. A. Peyser, L. F. Bielak, T. D. O'Connor, L. S. Emery; NHLBI Trans-Omics for Precision Medicine (TOPMed) Consortium; TOPMed Population Genetics Working Group, C. Gilissen, W. S. W. Wong, P. V. Kharchenko, S. Sunyaev, Population sequencing data reveal a compendium of mutational processes in the human germ line. *Science* **373**, 1030–1035 (2021).

46. I. Agarwal, M. Przeworski, Signatures of replication timing, recombination, and sex in the spectrum of rare variants on the human X chromosome and autosomes. *Proc. Natl. Acad. Sci. U.S.A.* **116**, 17916–17924 (2019).

47. F. Massip, M. Laurent, C. Brossas, J. M. Fernández-Justel, M. Gómez, M. N. Prioleau, L. Duret, F. Picard, Evolution of replication origins in vertebrate genomes: Rapid turnover despite selective constraints. *Nucleic Acids Res.* **47**, 5114–5125 (2019).

48. M. Touchon, S. Nicolay, B. Audit, E. B. Brodie of Brodie , Y. d'Aubenton-Carafa, A. Arneodo, C. Thermes, Replication-associated strand asymmetries in mammalian genomes: Toward detection of replication origins. *Proc. Natl. Acad. Sci. U.S.A.* **102**, 9836–9841 (2005).

49. J. Mrázek, S. Karlin, Strand compositional asymmetry in bacterial and large viral genomes. *Proc. Natl. Acad. Sci. U.S.A.* **95**, 3720–3725 (1998).

50. The FANTOM Consortium and the RIKEN PMI and CLST (DGT, A promoter-level mammalian expression atlas. *Nature* **507**, 462–470 (2014).

51. E. T. Wang, R. Sandberg, S. Luo, I. Khrebtukova, L. Zhang, C. Mayr, S. F. Kingsmore, G. P. Schroth, C. B. Burge, Alternative isoform regulation in human tissue transcriptomes. *Nature* **456**, 470–476 (2008).

52. M. Macheret, T. D. Halazonetis, Intragenic origins due to short G1 phases underlie oncogene-induced DNA replication stress. *Nature* **555**, 112–116 (2018).

53. M. Lawrence, R. Gentleman, V. Carey, rtracklayer: An R package for interfacing with genome browsers. *Bioinformatics* **25**, 1841–1842 (2009).

54. S. Durinck, P. T. Spellman, E. Birney, W. Huber, Mapping identifiers for the integration of genomic datasets with the R/Bioconductor package biomaRt. *Nat. Protoc.* **4**, 1184–1191 (2009).

55. A. R. Quinlan, I. M. Hall, BEDTools: A flexible suite of utilities for comparing genomic features. *Bioinformatics* **26**, 841–842 (2010).

56. R. S. Hansen, S. Thomas, R. Sandstrom, T. K. Canfield, R. E. Thurman, M. Weaver, M. O. Dorschner, S. M. Gartler, J. A. Stamatoyannopoulos, Sequencing newly replicated DNA reveals widespread plasticity in human replication timing. *Proc. Natl. Acad. Sci. U.S.A.* **107**, 139–144 (2010).

57. G. Zhao, K. Li, B. Li, Z. Wang, Z. Fang, X. Wang, Y. Zhang, T. Luo, Q. Zhou, L. Wang, Y. Xie, Y. Wang, Q. Chen, L. Xia, Y. Tang, B. Tang, K. Xia, J. Li, Gene4Denovo: An integrated database and analytic platform for de novo mutations in humans. *Nucleic Acids Res.* **48**, D913–D926 (2020).

58. P. Danecek, A. Auton, G. Abecasis, C. A. Albers, E. Banks, M. A. DePristo, R. E. Handsaker, G. Lunter, G. T. Marth, S. T. Sherry, G. McVean, R. Durbin; 1000 Genomes Project Analysis Group, The variant call format and VCFtools. *Bioinformatics* **27**, 2156–2158 (2011).

59. R. Gaujoux, C. Seoighe, A flexible R package for nonnegative matrix factorization. *BMC Bioinformatics* **11**, 367 (2010).

60. J. D. Buenrostro, B. Wu, H. Y. Chang, W. J. Greenleaf, ATAC-seq: A method for assaying chromatin accessibility genome-wide. *Curr. Protoc. Mol. Biol.* **109**, 21.29.1–21.29.9 (2015).

61. J. D. Buenrostro, P. G. Giresi, L. C. Zaba, H. Y. Chang, W. J. Greenleaf, Transposition of native chromatin for fast and sensitive epigenomic profiling of open chromatin, DNA-binding proteins and nucleosome position. *Nat. Methods* **10**, 1213–1218 (2013).

62. M. Martin, Cutadapt removes adapter sequences from high-throughput sequencing reads. *EMBnet J.* **17**, 10–12 (2011).

63. B. Langmead, S. L. Salzberg, Fast gapped-read alignment with Bowtie 2. *Nat. Methods* **9**, 357–359 (2012).

64. D. Kim, G. Pertea, C. Trapnell, H. Pimentel, R. Kelley, S. L. Salzberg, TopHat2: Accurate alignment of transcriptomes in the presence of insertions, deletions and gene fusions. *Genome Biol.* **14**, R36 (2013).

65. C. Trapnell, B. A. Williams, G. Pertea, A. Mortazavi, G. Kwan, M. J. van Baren, S. L. Salzberg, B. J. Wold, L. Pachter, Transcript assembly and quantification by RNA-Seq reveals unannotated transcripts and isoform switching during cell differentiation. *Nat. Biotechnol.* **28**, 511–515 (2010).

66. G. Tan, B. Lenhard, TFBSTools: An R/bioconductor package for transcription factor binding site analysis. *Bioinformatics* **32**, 1555–1556 (2016).

67. O. Fornes, J. A. Castro-Mondragon, A. Khan, R. van der Lee, X. Zhang, P. A. Richmond, B. P. Modi, S. Correard, M. Gheorghe, D. Baranašić, W. Santana-Garcia, G. Tan, J. Chèneby, B. Ballester, F. Parcy, A. Sandelin, B. Lenhard, W. W. Wasserman, A. Mathelier, JASPAR 2020: Update of the open-access database of transcription factor binding profiles. *Nucleic Acids Res.* **48**, D87–D92 (2020).

68. D. Charif, J. Lobry, SeqinR 1.0-2: A contributed package to the R project for statistical computing devoted to biological sequences retrieval and analysis, in *Structural Approaches to Sequence Evolution. Biological and Medical Physics, Biomedical Engineering*, U. Bastolla, M. Porto, H. E. Roman, M. Vendruscolo, Eds. (Springer, 2007), pp. 207–232.

69. H. Pagès, Aboyoun, P, R. Gentleman, S. DebRoy, Biostrings: Efficient manipulation of biological strings. R package version 2.60.2 (2021).

70. J. L. Huppert, S. Balasubramanian, Prevalence of quadruplexes in the human genome. *Nucleic Acids Res.* **33**, 2908–2916 (2005).

71. G. I. Dellino, D. Cittaro, R. Piccioni, L. Luzi, S. Banfi, S. Segalla, M. Cesaroni, R. Mendoza-Maldonado, M. Giacca, P. G. Pelicci, Genome-wide mapping of human DNA-replication origins: Levels of transcription at ORC1 sites regulate origin selection and replication timing. *Genome Res.* **23**, 1–11 (2013).

72. B. Miotto, Z. Ji, K. Struhl, Selectivity of ORC binding sites and the relation to replication timing, fragile sites, and deletions in cancers. *Proc. Natl. Acad. Sci. U.S.A.* **113**, E4810–E4819 (2016).

73. P. M. Martínez-García, M. García-Torres, F. Divina, J. Terrón-Bautista, I. Delgado-Sainz, F. Gómez-Vela, F. Cortés-Ledesma, Genome-wide prediction of topoisomerase IIβ binding by architectural factors and chromatin accessibility. *PLOS Comput. Biol.* **17**, e1007814 (2021).

74. G. I. Dellino, F. Palluzzi, A. M. Chiariello, R. Piccioni, S. Bianco, L. Furia, G. De Conti, B. A. M. Bouwman, G. Melloni, D. Guido, L. Giacò, L. Luzi, D. Cittaro, M. Faretta, M. Nicodemi, N. Crosetto, P. G. Pelicci, Release of paused RNA polymerase II at specific loci favors DNA double-strand-break formation and promotes cancer translocations. *Nat. Genet.* **51**, 1011–1023 (2019).

75. H. J. Gothe, B. A. M. Bouwman, E. G. Gusmao, R. Piccinno, G. Petrosino, S. Sayols, O. Drechsel, V. Minneker, N. Josipovic, A. Mizi, C. F. Nielsen, E. M. Wagner, S. Takeda, H. Sasanuma, D. F. Hudson, T. Kindler, L. Baranello, A. Papantonis, N. Crosetto, V. Roukos, Spatial chromosome folding and active transcription drive DNA Fragility and formation of oncogenic MLL translocations. *Mol. Cell* **75**, 267–283.e12 (2019).

76. A. Gardini, D. Baillat, M. Cesaroni, R. Shiekhattar, Genome-wide analysis reveals a role for BRCA1 and PALB2 in transcriptional co-activation. *EMBO J.* **33**, 890–905 (2014).

77. R. Xiao, J. Y. Chen, Z. Liang, D. Luo, G. Chen, Z. J. Lu, Y. Chen, B. Zhou, H. Li, X. Du, Y. Yang, M. San, X. Wei, W. Liu, E. Lécuyer, B. R. Graveley, G. W. Yeo, C. B. Burge, M. Q. Zhang, Y. Zhou, X. D. Fu, Pervasive chromatin-RNA binding protein interactions enable rna-based regulation of transcription. *Cell* **178**, 107–121.e18 (2019).

78. L. Baranello, D. Wojtowicz, K. Cui, B. N. Devaiah, H. J. Chung, K. Y. Chan-Salis, R. Guha, K. Wilson, X. Zhang, H. Zhang, J. Piotrowski, C. J. Thomas, D. S. Singer, B. F. Pugh, Y. Pommier, T. M. Przytycka, F. Kouzine, B. A. Lewis, K. Zhao, D. Levens, RNA polymerase II regulates topoisomerase 1 activity to favor efficient transcription. *Cell* **165**, 357–371 (2016).

79. N. Nalabothula, T. Al-jumaily, A. M. Eteleeb, R. M. Flight, S. Xiaorong, H. Moseley, E. C. Rouchka, Y. N. Fondufe-Mittendorf, Genome-wide profiling of PARP1 reveals an interplay with gene regulatory regions and DNA methylation. *PLOS ONE* **10**, e0135410 (2015).

80. H. Zhang, H. Gan, Z. Wang, J. H. Lee, H. Zhou, T. Ordog, M. S. Wold, M. Ljungman, Z. Zhang, RPA interacts with HIRA and regulates H3.3 deposition at gene regulatory elements in mammalian cells. *Mol. Cell* **65**, 272–284 (2017).

81. A. Promonet, I. Padioleau, Y. Liu, L. Sanz, A. Biernacka, A. L. Schmitz, M. Skrzypczak, A. Sarrazin, C. Mettling, M. Rowicka, K. Ginalski, F. Chedin, C. L. Chen, Y. L. Lin, P. Pasero, Topoisomerase 1 prevents replication stress at R-loop-enriched transcription termination sites. *Nat. Commun.* **11**, 3940 (2020).
